# Range-dependent thresholds for global flood early warning

**DOI:** 10.1016/j.hydroa.2019.100034

**Published:** 2019-07

**Authors:** Lorenzo Alfieri, Ervin Zsoter, Shaun Harrigan, Feyera Aga Hirpa, Christophe Lavaysse, Christel Prudhomme, Peter Salamon

**Affiliations:** a European Commission, Joint Research Centre (JRC), Ispra, Italy; b European Centre for Medium-Range Weather Forecasts (ECMWF), Reading, UK; c Oxford University, Oxford, UK; d University of Grenoble Alpes, CNRS, IRD, Grenoble, France

**Keywords:** Flood thresholds, Early warning system, GloFAS, Ensemble forecasting, Hydrologic model, Global hydrology

## Abstract

Early warning systems (EWS) for river flooding are strategic tools for effective disaster risk management in many world regions. When driven by ensemble Numerical Weather Predictions (NWP), flood EWS can provide skillful streamflow forecasts beyond the monthly time scale in large river basins. Yet, effective flood detection is challenged by accurate estimation of warning thresholds that identify specific hazard levels along the entire river network and forecast horizon. This research describes a novel approach to estimate warning thresholds which retain statistical consistency with the operational forecasts at all lead times. The procedure is developed in the context of the Global Flood Awareness System (GloFAS). A 21-year forecast-consistent dataset is used to derive thresholds with global coverage and forecast range up to six weeks. These are compared with thresholds derived from ERA5, a state of the art atmospheric reanalysis used to run the baseline simulation for the years 1986–2017 and to give a best guess of the present hydrological states. Findings show that the use of constant thresholds for 30-day flood forecasting, as in the current operational GloFAS setup, is consistent throughout the entire forecast range in only 30% to 40% of the river network, depending on the flood return period. Findings show that range-dependent thresholds, of weekly duration, are a more suitable alternative to time-invariant thresholds, as they improve the model consistency as well as the skills in flood monitoring and early warning, particularly over longer forecasting range.

## Highlights

• We present a novel approach to estimate flood warning thresholds in GloFAS.
• It preserve statistical consistency between thresholds and operational forecasts.
• Time-invariant thresholds are consistent with 6-week forecasts only in 27% of points.
• Range-dependent thresholds are a better alternative to time-invariant thresholds.

## 1 Introduction

Flooding is among the costliest disasters worldwide, with reported population affected of 80–110 million people per year (Jonkman, 2005, UNISDR and CRED, 2015) and flood losses estimated at 30–100 billion USD per year (Re, 2015, UNISDR, 2015, UNISDR and CRED, 2015). These figures are projected to rise even further in the coming decades due to socio-economic growth and the consequent increase in flood exposure and to ongoing global warming, which is linked to an intensification of weather-related disasters in most world regions (Dottori et al., 2018, Winsemius et al., 2016). Flood forecasting and early warning systems are cost-effective measures to reduce the impacts of floods by providing timely information on where and when floods will occur in the near future (Pappenberger et al., 2015a). A common approach to forecast floods in the medium range, i.e., 3–15 days ahead of their occurrence, is by forcing a hydrological model with an ensemble of numerical weather predictions (NWP) to estimate the probable future hydrological conditions (Cloke and Pappenberger, 2009, Fan et al., 2014, Pagano et al., 2014, Siddique and Mejia, 2017, Thielen et al., 2009, Thiemig et al., 2010). In addition, thanks to continuous improvements in weather prediction and to the long travel time of flood waves in the largest world’s rivers, large scale ensemble flood forecasting is pushing forward the limits of predictability by enabling skillful quantitative forecasts over longer forecast horizons.

The Global Flood Awareness System (GloFAS), is a global hydrological modeling and forecasting system jointly developed by the European Commission (EC) and the European Centre for Medium-Range Weather Forecasts (ECMWF), and is an operational system of the Copernicus Emergency Management Service since 2018. GloFAS is designed to forecast large scale river flooding worldwide by detecting river reaches where predicted streamflow has significant probability to exceed model-consistent warning thresholds in a 30-day forecasting horizon (Alfieri et al., 2013). The estimation of suitable exceedance thresholds is a key task in flood early warning, where alerts are determined by the ratio between streamflow estimates and reference thresholds. Hence, model consistency is achieved through the use of the same hydrological model and meteorological product to derive both streamflow forecasts and the reanalysis dataset used to derive the thresholds. Accurate representation of the true river conditions along the river network is a long standing challenge in the global hydrological community (Bierkens et al., 2015, Nijssen et al., 2001). Yet, several examples have shown that skillful early warning systems based on threshold exceedance analysis favor the concept of model consistency rather than a mere pursuit of the true absolute hydrological states (Pappenberger et al., 2015b, Raynaud et al., 2015, Reed et al., 2007).

In GloFAS, the estimation of warning thresholds is determined by the global meteorological datasets used to reproduce a long term streamflow simulation, and in turn derive threshold values for selected occurrence probabilities. The current operational setup stems from the outcomes of the work by Hirpa et al. (2016). The key question in Hirpa et al. (2016) is whether flood thresholds derived from a reforecast climatology, hence favoring the consistence between thresholds and daily operational forecasts, yield more skillful prediction of severe flood events compared to using thresholds derived from a reanalysis product, hence more focused on reproducing the true state of the atmosphere. To this end, the authors produced a continuous atmospheric forcing by concatenating data from the first days of a weather reforecasts dataset issued twice per week over 20 years, and found a marginal improvement in flood detection skills. The debate around the best choice of flood thresholds has been recently reopened, the main reasons being summarized as follow:

- ERA5, the latest generation global atmospheric reanalysis product by ECMWF, has become available in 2018 (Hersbach et al., 2018). Key features compared to the previous version ERA-Interim (Dee et al., 2011) include 10 years’ worth of advances in NWP, higher spatial resolution, improvements in the ingested observations, and a near-real time updating service (2–5 days latency) known as ERA5T, which makes it particularly appealing for the daily updating of the global hydrological conditions used to initialize GloFAS streamflow forecasts. Also, ERA5T is used for the initialization of the current operational GloFAS forecasts since November 2018.
- The approach currently used to produce a continuous meteorological climatology based on reforecasts generates streamflow statistics which are not fully consistent with those of the operational forecasts, hence possibly originating a bias in the resulting flood warning thresholds.
- The extension in 2018 of GloFAS products to a 30-day forecast horizon urged the need to investigate whether thresholds derived from short-term forecasts (i.e., with lead time up to 4 days, see Hirpa et al., 2018) are suitable over a longer forecasting range.

Hence, this research effort was conducted to address the above issues with the following objectives:

1. Improving the methodology to estimate GloFAS warning thresholds, to make them statistically consistent with real-time streamflow forecasts along the entire forecast range.
2. Assessing the variability of flood thresholds through the forecast range.
3. Assessing whether the approach based on time-invariant flood thresholds is fit for purpose or whether range-dependent thresholds should be adopted instead through the forecast horizon.

In this article we present a new approach for deriving GloFAS flood thresholds over different weekly forecast horizons, based on an atmospheric reforecasts dataset, and we compare them with thresholds based on a global hydrological reanalysis forced by ERA5, hence consistent with the initial hydrological conditions.

## 2 Material and methods

### 2.1 Meteorological data

#### 2.1.1 ERA5

ERA5 (Hersbach et al., 2018) is the latest climate reanalysis dataset produced by ECMWF through the Copernicus Climate Change Service (C3S). ERA5 data extending from 1979 to the present became available at the end of 2018, while the second phase, extending back to 1950, is planned for release by autumn 2019. ERA5 is based on the Integrated Forecasting System (IFS) Cycle 41r2 which was operational in 2016. Therefore, ERA5 benefits from a decade of developments in model physics, numerics and data assimilation, compared to the ERA-Interim IFS Cycle 31r2, operational in 2006. In addition to a significantly enhanced horizontal resolution of 31 km, compared to 80 km for ERA-Interim, ERA5 has a number of innovative features, including hourly output, and an uncertainty estimate. ECMWF forecasts from ERA5 analysis show a gain of up to one day in skill with respect to ERA-Interim (Haiden et al., 2018), which is reflected not only in average weather variables but also with regard to large-scale weather patterns such as tropical cyclones.

In this work we used daily maps of precipitation, minimum, mean and maximum surface air temperature, mean sea level pressure, incoming solar radiation, wind speed and relative humidity extracted from ERA5 for 32 complete years between 1986 and 2017. Some variables were then processed to produce estimates of potential evapotranspiration using the Penman-Monteith equation. This represents one of the main dynamic input variables of the hydrological model Lisflood, together with mean surface air temperature and precipitation. ERA5 data was used to model the daily hydrological states over 1986–2017.

#### 2.1.2 Atmospheric reforecasts

ECMWF reforecasts are global scale forecast runs that use the same Integrated Forecasting System (IFS) model version as the real-time ensemble forecasts (ECMWF-ENS), for the past 20 years. Similar to the operational forecasts, reforecasts have horizontal resolutions of 18 km for up to 15-day lead time and 36 km for longer forecast lead times up to 46-day. In this work we used the unperturbed ensemble member (i.e., the control run) of 6-week reforecasts with daily resolution, initialized once per week in the entire period of availability, coupled with the last year of operational forecasts. The collected dataset thus includes 1218 sets of reforecasts with global coverage, spanning 21 years between 21/11/1996 and 25/12/2017. More in details, each of the 21 years of data is composed by 58 weekly reforecasts starting in mid-November of the previous year, so that each day of each year is ultimately simulated in 6 different reforecasts with lead time between 1 and 6 weeks. As for ERA5, we extracted the same set of atmospheric variables and pre-processed them to obtain daily estimates of evapotranspiration, which were used as input of the hydrological model together with mean surface air temperature and precipitation.

### 2.2 Hydrological modeling

Hydrological simulations are performed with Lisflood (van der Knijff et al., 2010), a distributed semi-physically based model developed at the Joint Research Centre of the European Commission. Processes simulated by Lisflood include soil freezing, snowmelt, surface runoff, infiltration, preferential flow, redistribution of soil moisture within the soil profile, drainage to the groundwater system, groundwater storage, and base flow. Runoff is produced at every grid cell and routed through the river network using a kinematic wave approach. Lisflood underpins a number of large scale applications ranging from climate change studies (Alfieri et al., 2015, Dankers and Feyen, 2009, Rojas et al., 2012), flood risk assessments (Alfieri et al., 2016), operational flood early warning systems (Thielen et al., 2009) and flood hazard mapping (Alfieri et al., 2014, Dottori et al., 2016). In this work, we used a global setup at 0.1 degree and daily resolutions, covering the Earth’s land areas except Iceland, Greenland and Antarctica. The global setup of the model was calibrated against observed daily streamflow for 24 large river basins (84,000–4,680,000 km^2^ in area) across the globe using the WFDEI (Weedon et al., 2014) atmospheric forcing and a multi-objective function incorporating bias, Nash-Sutcliffe efficiency (NSE), and log-transformed NSE (Beck et al., 2017). The calibrated river basins cover 17% of the entire simulation area, while for the uncalibrated area we used a common parameter set derived by expert judgement. We expect the model calibration to have a relatively minor impact on the outcomes of this work, as all simulations are run with the same model setup and their differences mostly depend on differences in the weather forcing and in their statistics, rather than on skills versus measured discharges. It is worth noting that the hydrological model used in this work is different from the setup used in the operational GloFAS, which is based on a combination of two models for the land surface and for the river routing component, respectively (see Hirpa et al., 2018).

### 2.3 Methods

We investigated differences in the statistics between ERA5 and the reforecasts dataset at all lead times, and their resulting hydrological simulations. The analysis is split in two steps, first focusing on the precipitation statistics and then on the flood warning thresholds. With regard to precipitation, we analyzed the mean annual maxima of daily precipitation (Pmax) and the mean annual precipitation (MAP), including spatial patterns of the differences between the two datasets. Due to their non-parametric nature, precipitation statistics are key diagnostic indicators that anticipate and explain possible differences between the corresponding hydrological model outputs. In this regard, Pmax is representative of extreme precipitation and is generally well correlated to floods (e.g., Guillot and Duband, 1967), while MAP is an indicator of long term bias between the considered datasets.

The second part of the analysis is performed on flood thresholds based on ERA5 versus those based on reforecasts. To this end, we first ran a 32-year global hydrological simulation forced with ERA5 and extracted discharge time series at all points of the river network. Then, 1,218 sets of 6-week reforecasts, initialized once per week between 21/11/1996 and 25/12/2017, were used as input to run as many hydrological simulations, taking initial conditions from the corresponding date in the ERA5-based run. Such model runs forced by reforecasts include 58 different 6-week simulations per year, starting on the 21 November of the previous year, so that each day of each year is ultimately modeled in six different simulations, with forecast range spanning between 1 and 6 weeks. We applied Extreme Value Distribution (EVD) fitting to the annual maximum discharges extracted from the ERA5-based dataset as well as from the six datasets stemming from reforecasts, after reordering the data by weekly lead time. The first two years of the ERA5-based dataset were discarded to guarantee sufficient model warm-up, leaving 30 years of data for the EVD fitting, while in the case of reforecasts all 21 years of data were used, given that meaningful initial conditions were provided by the ERA5-based run. Such 9-year difference (i.e., 30 versus 21 years) in the length of the two datasets may produce discontinuities in the related flood thresholds, in case of substantial differences in the statistics of the extremes in those 9 years when reforecasts are not available. In view of the direct implications of this research with the operational runs of GloFAS we have opted to accept this potential discontinuities, for the following reasons:

• While the ERA5 dataset is continuously extended in near-real time and therefore keeps growing in size, ECMWF reforecasts are regenerated every week using the latest IFS model version (which is updated more than once per year), yet only for the most recent 20 years. It follows that this 9-year gap will not be closed in the future unless a longer reference period is chosen at ECMWF for issuing reforecasts.
• 30 years are a commonly accepted duration for a reference climate, as defined by the World Meteorological Organization, as well as a recommended standard for extreme value analysis (e.g., Burroughs, 2003), hence it should be a preferred duration for use whenever available.

The chosen EVD fitting is based on the Gumbel distribution with the method of L-Moments (Hosking, 1990), an effective and parsimonious choice which shows best performance for relatively small sample sizes (Cunnane, 1989) as in this case. Significance of the analytical curves was assessed by bootstrapping, using 1000 repetitions for each fit. 90% confidence bands between the 5th and 95th percentile around the central estimates were extracted and used to assess the fitting uncertainty.

**Fig. 1:**
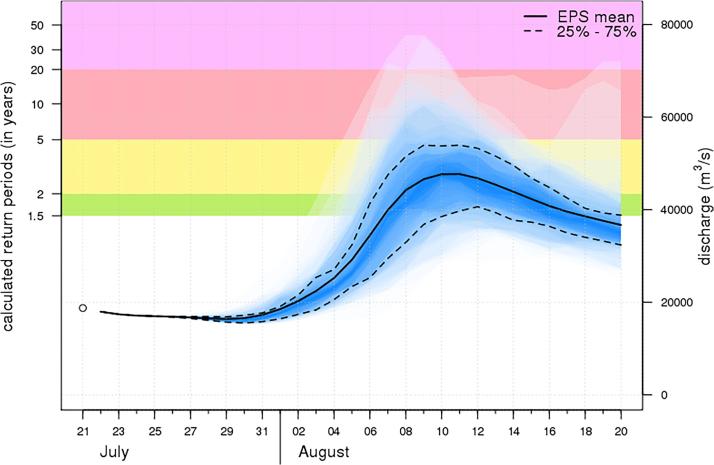
30-day GloFAS ensemble forecasts issued on 21/7/2018 for the Ganges at Hardinge Bridge (Bangladesh) and flood warning thresholds (color stripes) for return periods of 1.5, 2, 5 and 20 years (from http://www.globalfloods.eu).

In the remainder, we refer to flood thresholds as all discharge values estimated from analytical extreme value distributions, corresponding to return periods equal or larger than 2 years. In reality, the occurrence of actual flooding depends on the flood protection standards along the river network, which are known to vary considerably from around 2 to several thousand years, depending on the exposure and on the country’s investment capacity (Scussolini et al., 2016). In the operational version of GloFAS, reference warning thresholds correspond to return periods of discharge peaks of 1.5, 2, 5 and 20 years (see Fig. 1), following the recommendations of the European Flood Awareness System (EFAS, see Bartholmes et al., 2009). The 20-year threshold values are considered the most representative for detecting flood alerts in GloFAS, hence a number of figures in the remainder show results for such return periods, while results for return periods of 2, 5, 10, 20, 50, 100, 200 and 500 years are shown in the Supplementary material for completeness. One should note that results for return periods larger than the number of years of the time series (i.e., 50, 100, 200 and 500 years) are likely to be affected by large uncertainty bounds, and by possible discrepancies in the behavior of the tail of the hypothesized distribution. Yet, it is valuable to analyze them as they could impact a number of ensuing applications focused on flood hazard mapping and impact assessment (Alfieri et al., 2017, Dottori et al., 2016).

## 3 Results

**Fig. 2:**
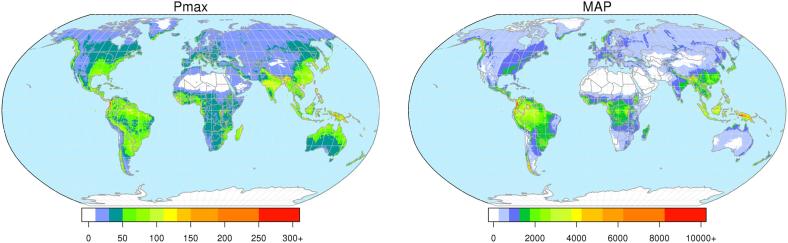
Mean annual maxima of daily precipitation (Pmax, left) and mean annual precipitation (MAP, right) from ERA5 (1986–2017) in mm.

**Fig. 3:**
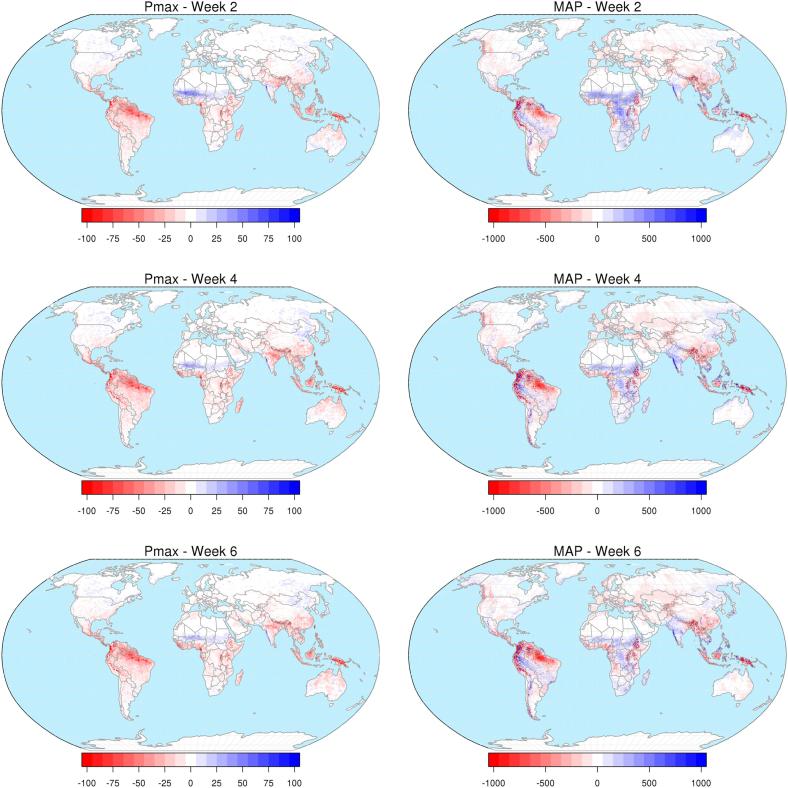
Maps of difference (in mm) in Pmax (left) and MAP (right) between reforecasts and ERA5, for forecast ranges of 2, 4 and 6 weeks.

Statistics of precipitation were calculated over the world’s land area using ERA5 (Fig. 2) and reforecasts with weekly lead time between 1 and 6 weeks. Maps of difference in Pmax and MAP between reforecasts and ERA5 are shown in Fig. 3, for forecast lead times of 2, 4, and 6 weeks. More in details, results for week 1 include all days with forecast lead time between 1 and 7 days, week 2 includes days with lead time between 8 and 14 days, and so forth up to week 6. Extreme daily precipitation simulated by reforecasts are smaller than those of ERA5 in most tropical and equatorial regions. Conversely, reforecasts simulate more extreme precipitation in the Sahel region, West India, parts of Canada and Eastern Asia. Mean annual precipitation shows a less homogeneous pattern, with reforecasts being above ERA5 in India, Central Africa and parts of South America, and below ERA5 in South-East Asia, Europe and parts of South America. No clear trends in the precipitation statistics appear along the forecast range, though the negative bias of reforecasts (both Pmax and MAP) tends to increase with the lead time for the majority of the land points. At the global scale, mean reforecasts-based MAP is smaller than the ERA5-based MAP by between 2.5 mm year^−1^ at week 1, and 5 mm year^−1^ at week 4, 5, and 6.

**Fig. 4:**
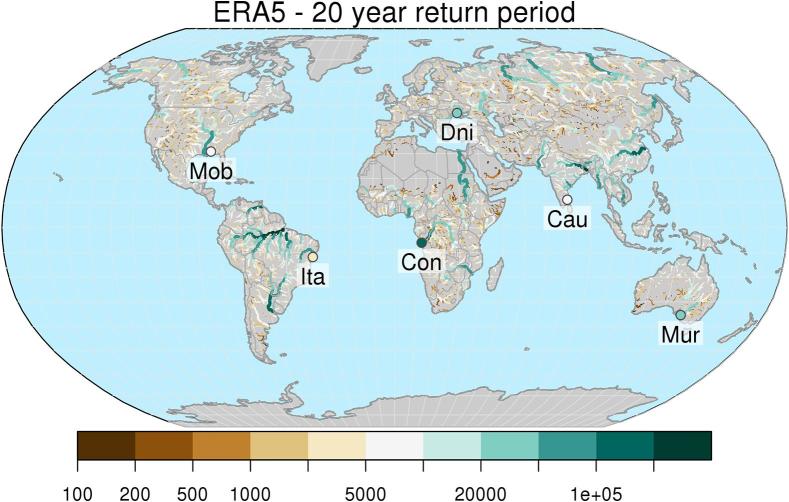
Map of peak discharges (in m^3^ s^−1^) with average return period of 20 years, using ERA5 as forcing. Circles indicate the outlet of the rivers Mobile (Mob), Itapicuru (Ita), Congo (Con), Dniepr (Dni), Cauvery (Cau), and Murray-Darling (Mur). Only river sections with upstream area larger than 10,000 km^2^ are shown, for easier interpretation of the plot.

**Fig. 5:**
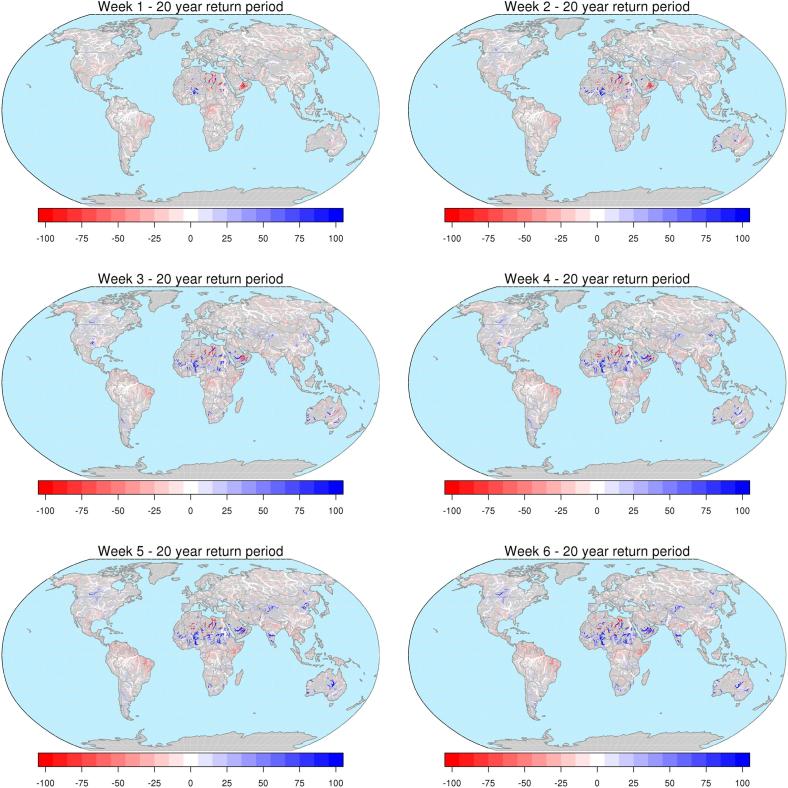
Maps of relative difference (in percent) in the 20-year threshold maps between reforecasts and ERA5, for forecast range between 1 and 6 weeks. Only river sections with upstream area larger than 10,000 km^2^ are shown, for easier interpretation of the plots.

**Fig. 6:**
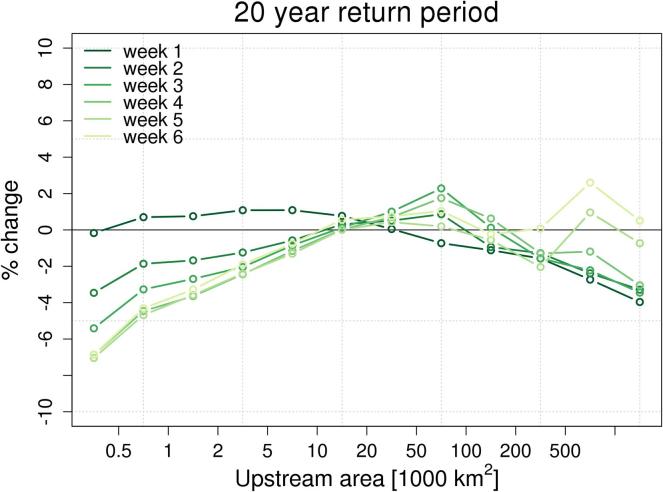
Relative difference (in percent) in the 20-year threshold values between reforecasts and ERA5, for forecast range between 1 and 6 weeks. Dots connected by lines show median values for each class of upstream area.

The map in Fig. 4 shows central estimates of maximum peak discharges with average recurrence interval of 20 years, based on the ERA5-driven simulation. Percent relative differences between reforecasts-based and ERA5-based maps are shown in Fig. 5 for lead-times between 1 and 6 weeks, together with a representation per upstream area classes in Fig. 6. Relative differences between reforecasts and ERA5 flood thresholds tend to increase in drier regions as well as with increasing forecast lead time. In larger rivers, differences are smaller and vary more gradually due to the larger influence of the initial hydrological conditions. Looking at changes across the range of considered flood magnitudes (see Supplement material, Figs. S4–S11), one can note a progressive reduction of flood thresholds derived from reforecasts compared to ERA5 from a return period of 2 years, moving towards 500 years. With regard to differences versus upstream area, the median change of each upstream class ranges within − 4% and + 1% at week 1, up to within − 7% and + 2% at week 6 (Fig. 6). When different quantiles of the distribution of the changes per upstream area classes are considered, we found an increasing spread of the empirical distribution of data for increasing values of forecast horizon and return period, and for decreasing values of upstream area (see Supplement material, Figs. S13–S20). At 20-year return period, the interquartile range (i.e., the central 50%) of the distribution of differences, ranges between − 10% and + 10% at week 1, up to − 21% and + 16% at week 6 (see Fig. S16).

**Fig. 7:**
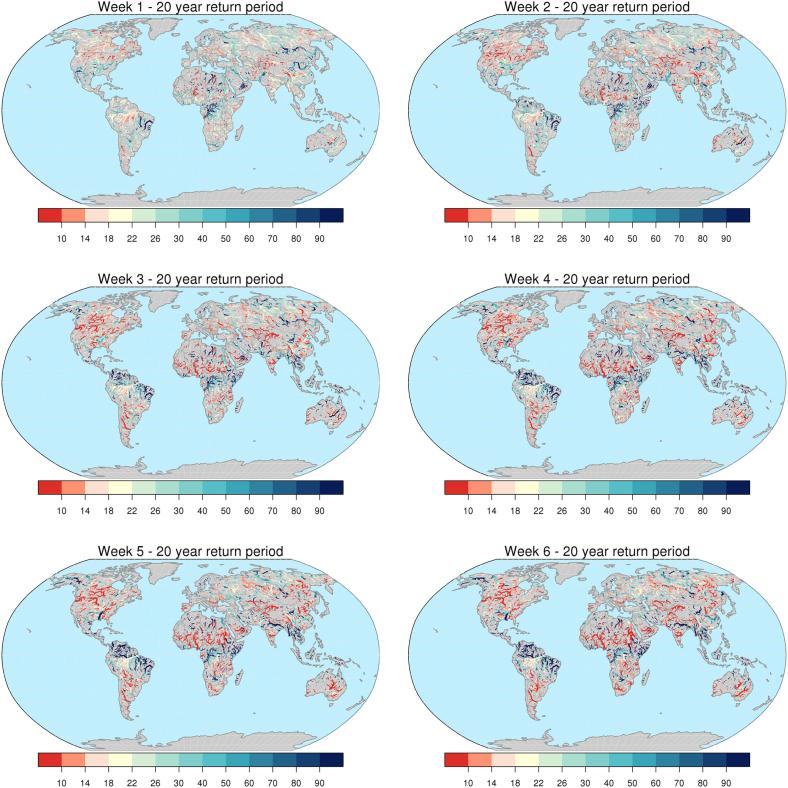
Return period (in years) of the ERA5 20-year discharge threshold map, obtained using the extreme value distributions of reforecasts-driven simulations, for forecast range between 1 and 6 weeks. Only river sections with upstream area larger than 10,000 km^2^ are shown, for easier interpretation of the plots.

**Fig. 8:**
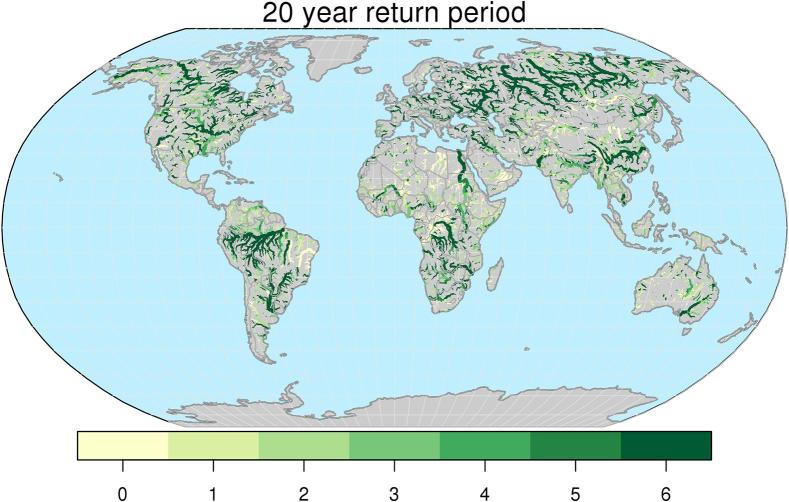
Number of weeks during which ERA5-based thresholds with 20-year return period are consistent with reforecast-based thresholds (i.e. within the 90% confidence bands).

**Fig. 9:**
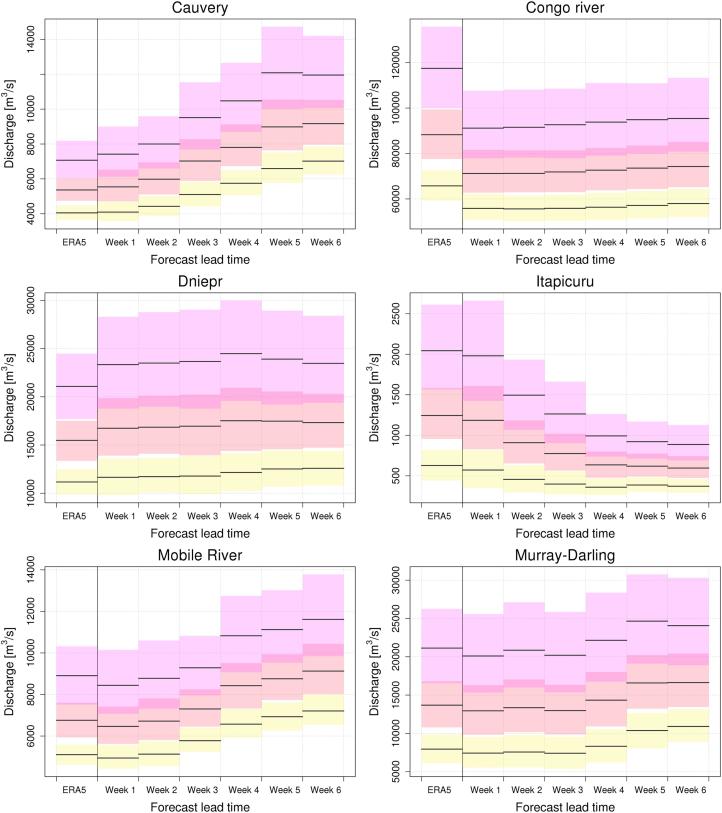
2, 5 and 20-year threshold values based on ERA5 and reforecasts (1–6 weeks lead time), at the outlet of six rivers (see location in Fig. 4). Central estimates (black horizontal lines) are shown together with the 90% confidence bands (yellow: 2 years, orange: 5 years, purple: 20 years). (For interpretation of the references to color in this figure legend, the reader is referred to the web version of this article.)

Differences in the thresholds described above, based on different hydrological simulations, cause bias and hence inappropriate interpretation of ERA5 flood thresholds when they are used in the prediction of threshold exceedances along the forecast range. Such bias can be evaluated by inverting the EVD of reforecasts at each weekly lead-time, using discharge values corresponding to flood thresholds based on the ERA5 EVD fitting. An example for a return period of 20 years is shown in Fig. 7, for lead times between 1 and 6 weeks. Light yellow pixels in the figure represent river points where a 20-year flood peak magnitude in the ERA5 simulation corresponds to the same return period magnitude (±10%) also in the reforecasts-based run. Conversely, shades of red (blue) indicate that 20-year discharges from ERA5 actually correspond to smaller (larger) return periods when used in predictive mode. 90% confidence intervals around central estimates of flood thresholds are used to assess statistically significant differences between flood thresholds stemming from different input datasets, and at different lead times. Hence, assuming a confidence interval of 90%, we show in Fig. 8 the number of subsequent weeks over which ERA5-based 20-year thresholds fall within the distribution of range specific thresholds derived from reforecasts. We found that only in 27% of grid points, are ERA5-based 20-year thresholds statistically consistent with the entire 6-week streamflow forecasts. On the other hand, in 44% of grid points ERA5-based 20-year thresholds are not consistent with those derived from reforecasts already from a forecast range of 1 week. The remaining 29% of grid points take on intermediate values between 1 and 5 weeks of consistency of the two sets of thresholds. Similar figures were found across the considered flood return periods (see Supplement material, Fig. S30). For instance, taking the current GloFAS configuration of ∼ 4-week forecasts, ERA5-based thresholds could be consistently used through the entire forecasting range only in 39–31% of GloFAS grid points, for return periods between 2 and 500 years respectively. A closer look is given in Fig. 9, which shows six examples of the proposed range-dependent flood thresholds evaluated at the outlet of six rivers, together with their 90% confidence intervals. The six cases were chosen to represent different continents, climate regions, basin size, as well as different trends in the threshold values along the 6-week forecast range. Among these six stations, 2 cases (Dniepr and Murray-Darling) retain low variability of flood thresholds over the entire 6-week range. Two cases (Cauvery and Mobile River) show a significantly rising trend, while two cases (Itapicuru and Congo River) a decreasing trend. An interesting example is that of the Congo River, where thresholds are almost constant through the entire forecast range, though they are significantly smaller than those derived from ERA5. Such differences are due to a number of extreme events simulated by ERA5 in 1986–1996, which are therefore not included in the shorter series derived from the reforecasts dataset, (i.e., 1997–2017), hence causing the type of discontinuity described in Section 2.3.

## 4 Discussion

Results of our analyses show a complex spatial pattern of sign and magnitude of the differences between thresholds derived from a reanalysis dataset (i.e., ERA5) and those derived from a forecast-consistent dataset (i.e., the reforecasts). Differences do not appear to have clear linear dependence with a number of considered variables, such as return period of the flood thresholds, weekly forecast range, latitude, basin upstream area and mean discharge. Instead the pattern of such differences follows more that of the differences in precipitation statistics (see Fig. 3 and 5), hence it is specific of the chosen atmospheric datasets. However, we found a tendency for larger discrepancies between ERA5- and reforecasts-based flood thresholds in smaller and drier river basins, over longer forecast ranges. Conversely, flood thresholds in large rivers over short forecast ranges often follow closely those based on ERA5, from which they take the initial conditions determining a large proportion of the river streamflow.

The findings of this research indicate that range-dependent thresholds should be used and replace time-invariant thresholds to improve the consistency in flood monitoring and early warning, especially over longer forecast ranges. Unlike the constant-thresholds setup, the proposed approach would retain consistency along the entire forecast range with the operational streamflow simulations, which integrate the effect of three IFS model configurations with different spatial resolution. First, the ERA5 atmospheric reanalysis with grid resolution of 31 km, used in the baseline run from which hydrological initial conditions are taken. Second, medium-range forecasts up to 15 days with grid resolution of 18 km, which are primarily governed by atmospheric initial values. Third, extended-range forecasts with ocean–atmosphere coupling from 16 to 46 days with grid resolution of 36 km, where the sea-surface temperature and associated heat and moisture start having non-negligible influence on the atmospheric evolution (Owens and Hewson, 2018).

Traditionally, flood thresholds used in short-term forecasting and early warning are based on observations or reconstructed extreme value distributions based on long term simulations (Pappenberger et al., 2015b). On the other hand long term (e.g., seasonal) forecasts use less extreme thresholds based on anomalies or percentiles from the streamflow distributions, to compensate for the smoothing effect of coarser resolution modeling, forecast uncertainty, temporal aggregation of results and variability of the precipitation statistics in the long-range forecasting (Arnal et al., 2018, Emerton et al., 2018). In this context, we see our approach as a step in the right direction for a smooth transition between quantitative thresholds of discharge or water level, to long range forecasts which are tailored more on the identification of wetter- or drier-than-normal periods. In an ideal seamless forecasting system, seasonal forecasts would anticipate wetter than normal periods, which could potentially become large-scale river flooding and be detected as severe events as early as a few weeks before, leaving enough time for low-cost preparatory actions. As the event approaches, short-term forecasts enable improved quantitative estimation of river discharges and corresponding water levels, hence transitioning to impact-based assessments including the exposure and vulnerability of the potentially affected areas, so to prioritize actions for an efficient emergency management.

To the authors’ knowledge, no operational system for medium-range flood early warning uses range-dependent thresholds to detect upcoming severe events. The concept of range-dependent thresholds may initially be difficult to accept for users, as it eliminates the univocal link between discharge thresholds and their probability of occurrences. In other words, any given discharge peak would correspond to a different probability of occurrence depending on when it is forecast in the future. Yet, the proposed approach is more correct and consistent with the data, hence it is likely to improve the estimation of the magnitude of upcoming extreme events over longer forecast ranges.

## 5 Conclusions

In this research we have tested a new method to estimate discharge thresholds for medium-range flood forecasting, for potential use in the Global Flood Awareness System. We have provided evidence that flood thresholds for medium-range forecasting can take on significantly different values from thresholds derived from the reference baseline run forced by ERA5 data, used to initialize the forecasts. In addition, thresholds often vary throughout consecutive weekly forecast ranges with a complex pattern of change, mostly driven by differences in the atmospheric datasets used as forcing to the hydrological model. Therefore, we propose a modified framework for the early detection of floods, based on range-dependent flood thresholds, which are able to compensate for forecast drifts in the extreme values, particularly useful over long ranges. Upcoming work, ahead of the operational implementation, will involve extensive testing and skill evaluation of the operational ensemble forecasts, to assess the limits of predictability of the system and compare the system skills to the current operational version. This represents a key step in the evolution of GloFAS which may ultimately lead to the extension from the current 30-day to a 6-week forecasting range. It is worth noting that flood thresholds based on reforecasts must be updated regularly as new IFS cycles are released, to retain consistency between streamflow predictions and warning thresholds and improve the detection of severe floods along the entire forecast horizon.

## Declaration of Competing Interest

The authors declare that they have no known competing financial interests or personal relationships that could have appeared to influence the work reported in this paper.
