# Supplementary material for: Range-dependent thresholds for global flood early warning
Source: J Hydrol X. 2019 Jul;4:100034. doi: 10.1016/j.hydroa.2019.100034 (PMC6894274; doi:10.1016/j.hydroa.2019.100034)
Supplement: Supplementary data 1 [file mmc1.docx]

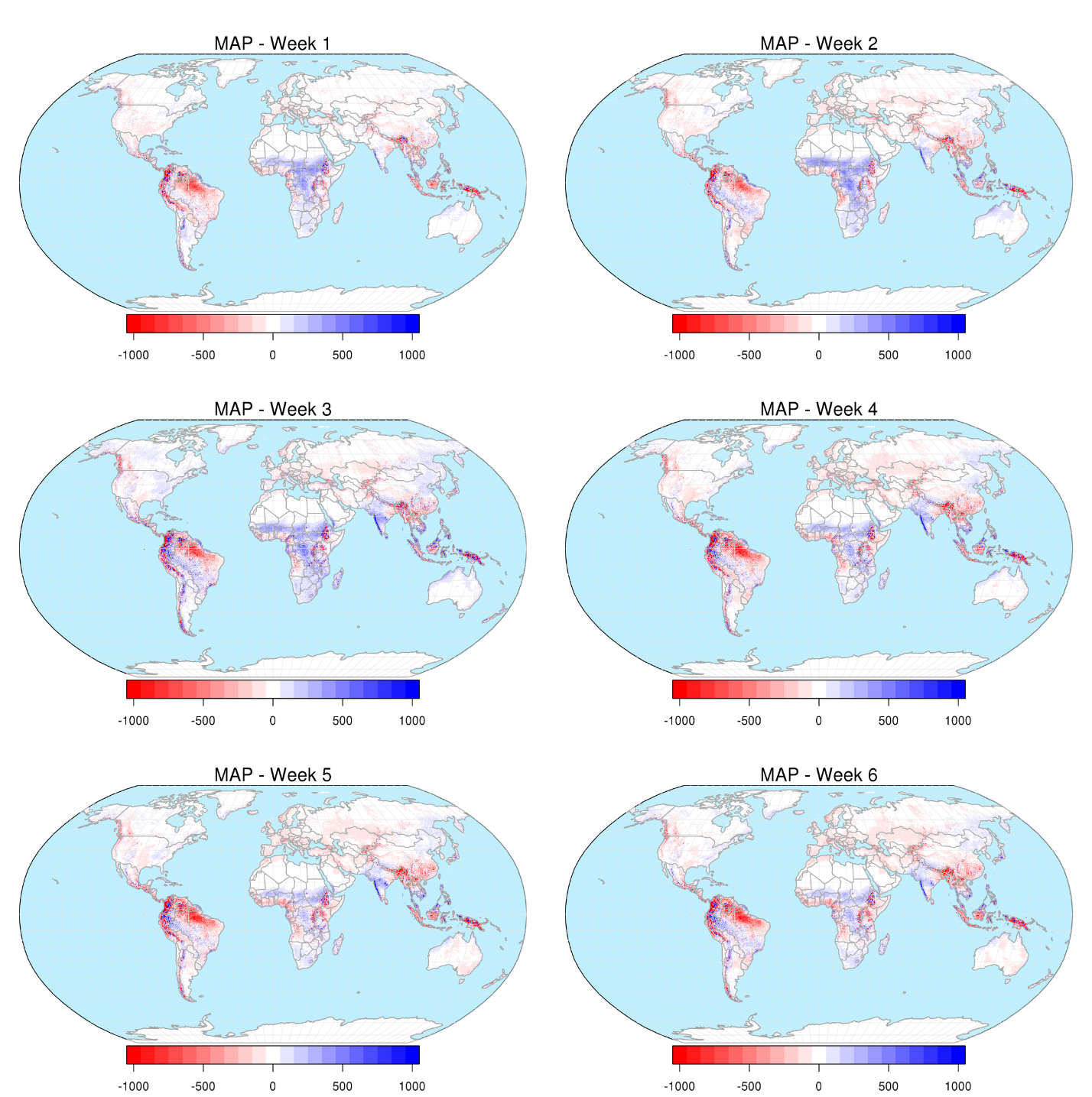


Figure S1: Maps of difference (in mm) in mean annual precipitation (MAP) between reforecasts and ERA5, for forecast ranges between 1 and 6 weeks.


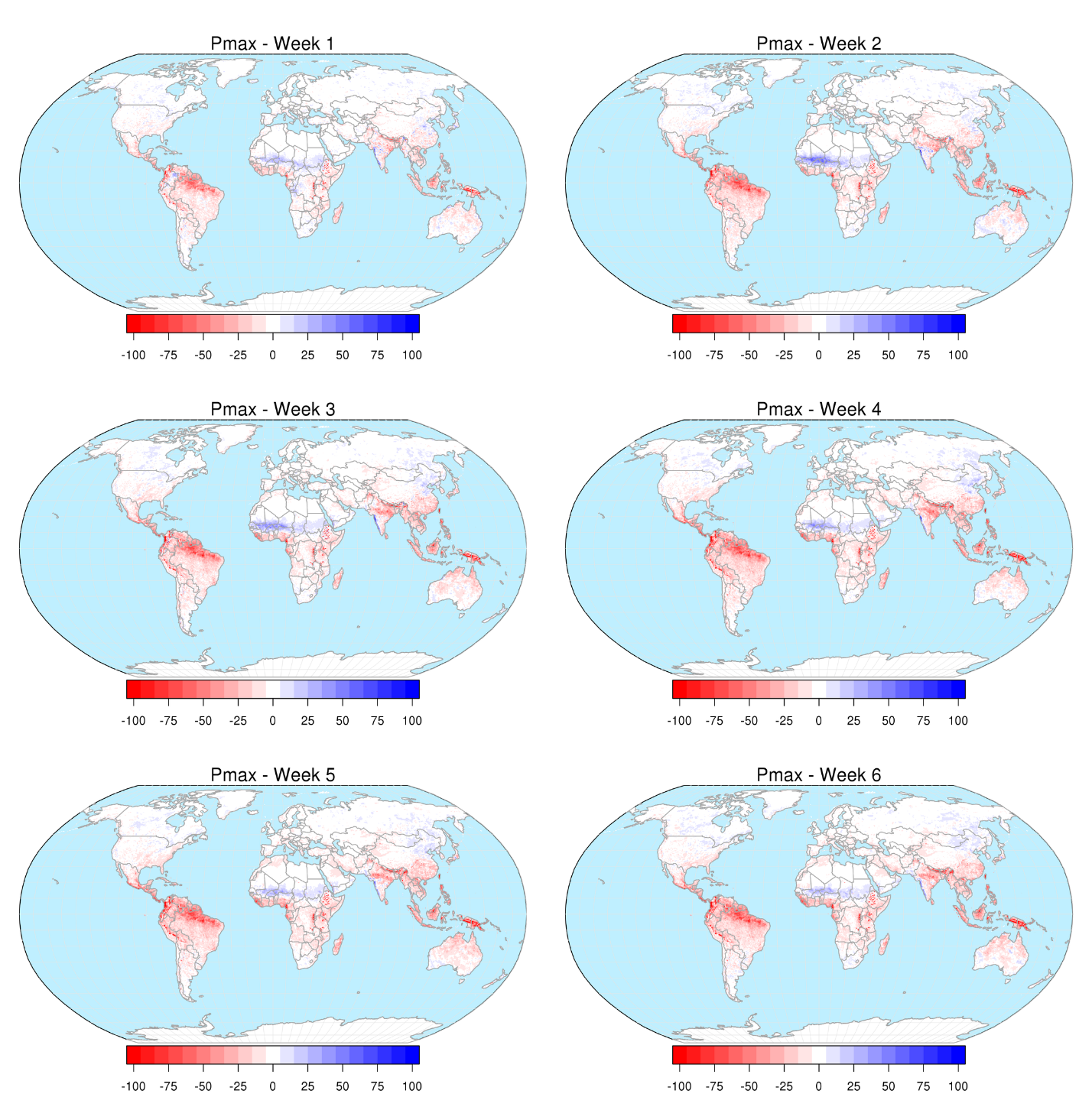


Figure S2: Maps of difference (in mm) in mean annual maxima of daily precipitation (Pmax) between reforecasts and ERA5, for forecast ranges between 1 and 6 weeks.


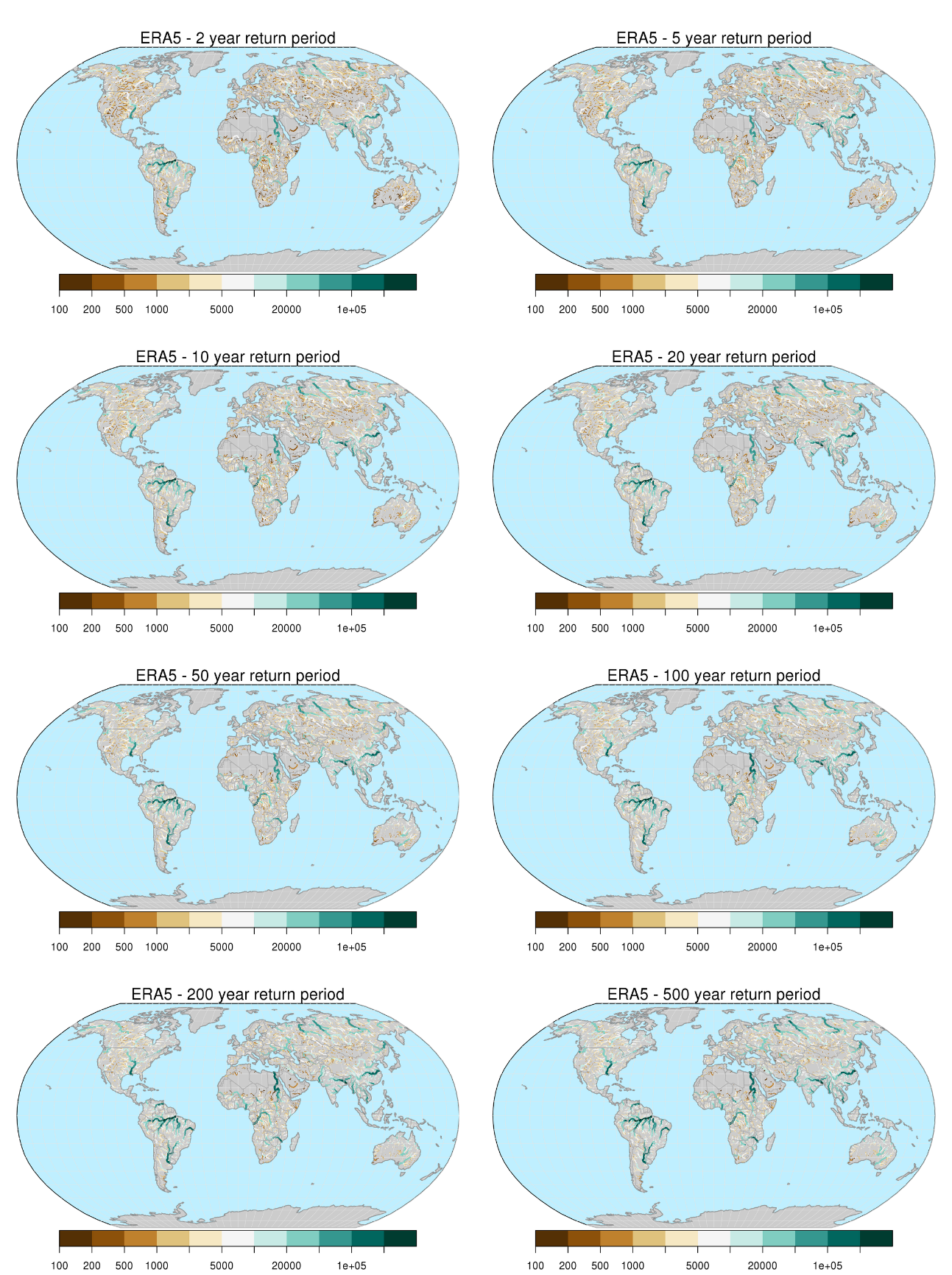


Figure S3: Map of peak discharges (in m^3^ s^-1^) with average return period of 20 years, using ERA5 as forcing. Only river sections with upstream area larger than 10,000 km^2^ are shown, for easier interpretation of the plot.


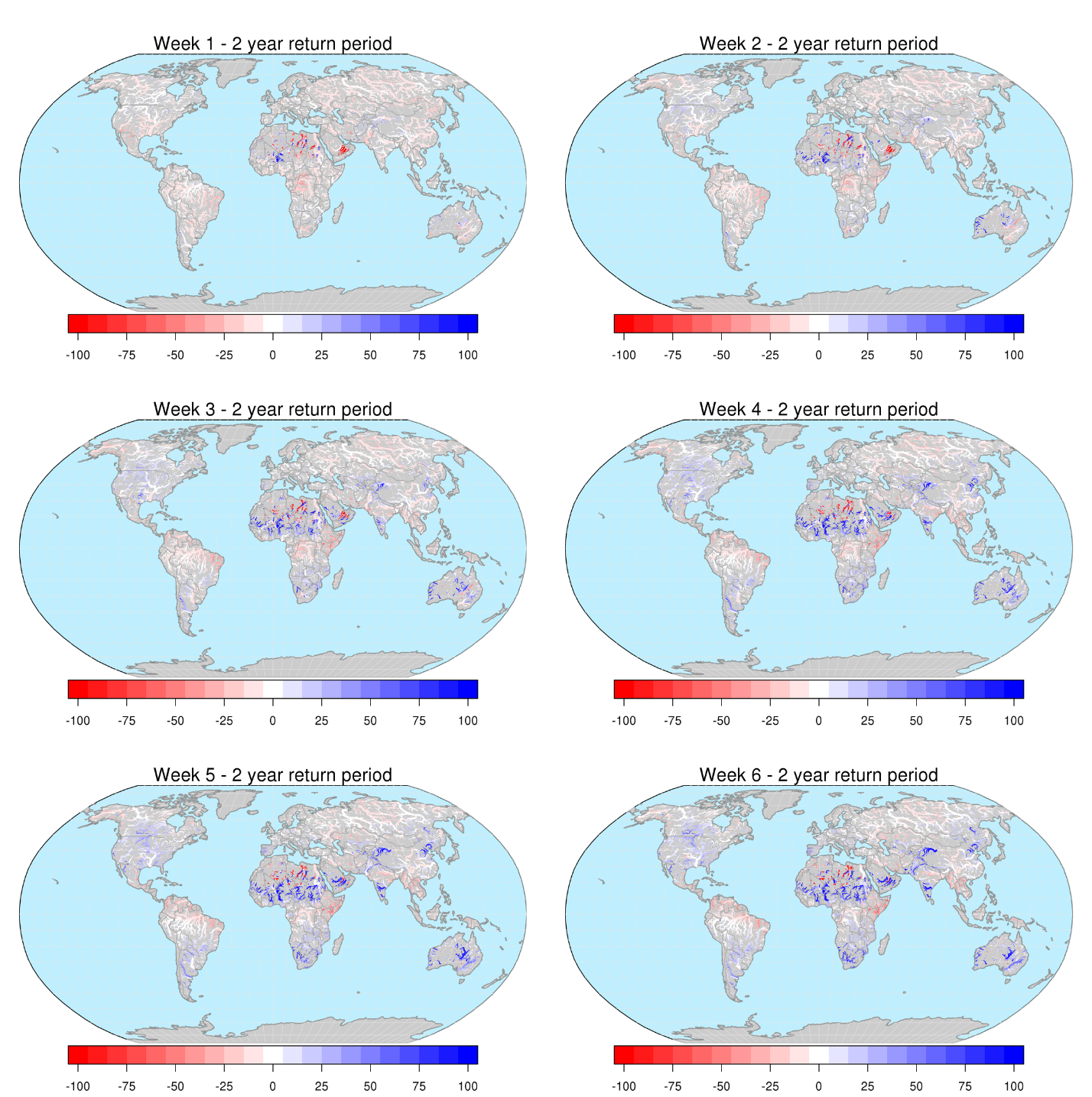
Figure S4: Relative difference (in percent) in the 2-year threshold maps between reforecasts and ERA5, for forecast range between 1 and 6 weeks. Only river sections with upstream area larger than 10,000 km^2^ are shown, for easier interpretation of the plots.
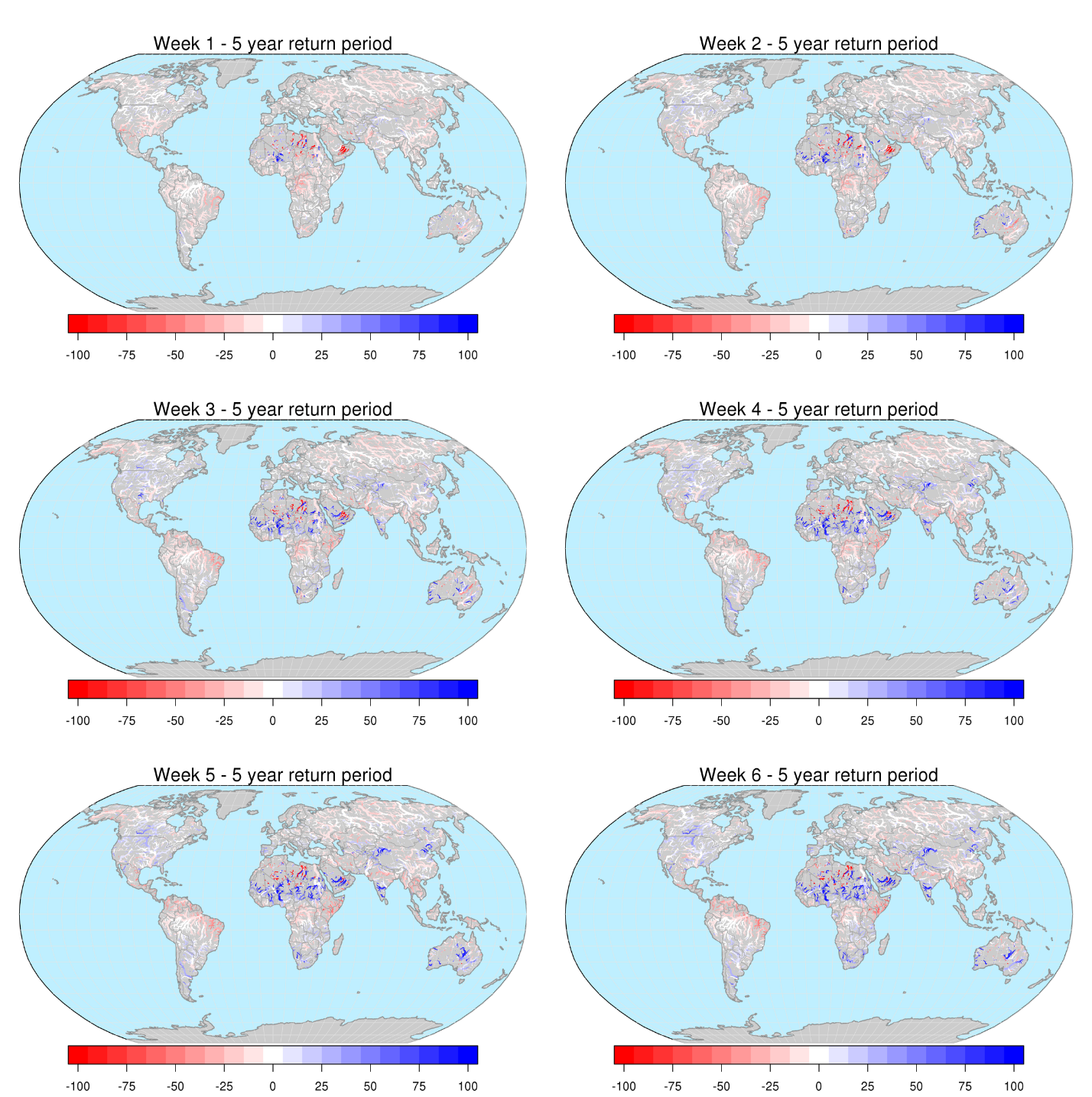
 Figure S5: Relative difference (in percent) in the 5-year threshold maps between reforecasts and ERA5, for forecast range between 1 and 6 weeks. Only river sections with upstream area larger than 10,000 km^2^ are shown, for easier interpretation of the plots.
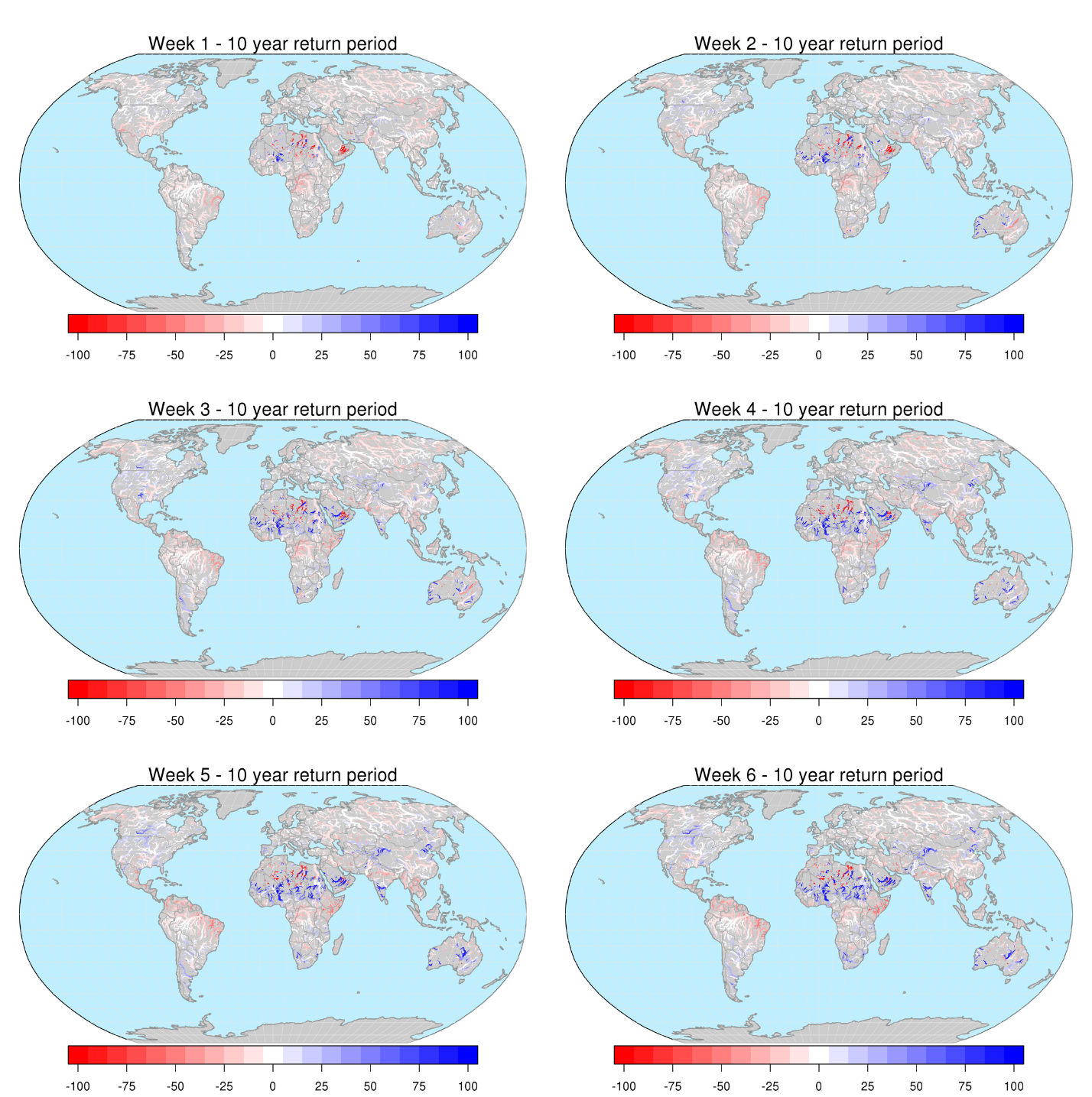
 Figure S6: Relative difference (in percent) in the 10-year threshold maps between reforecasts and ERA5, for forecast range between 1 and 6 weeks. Only river sections with upstream area larger than 10,000 km^2^ are shown, for easier interpretation of the plots.
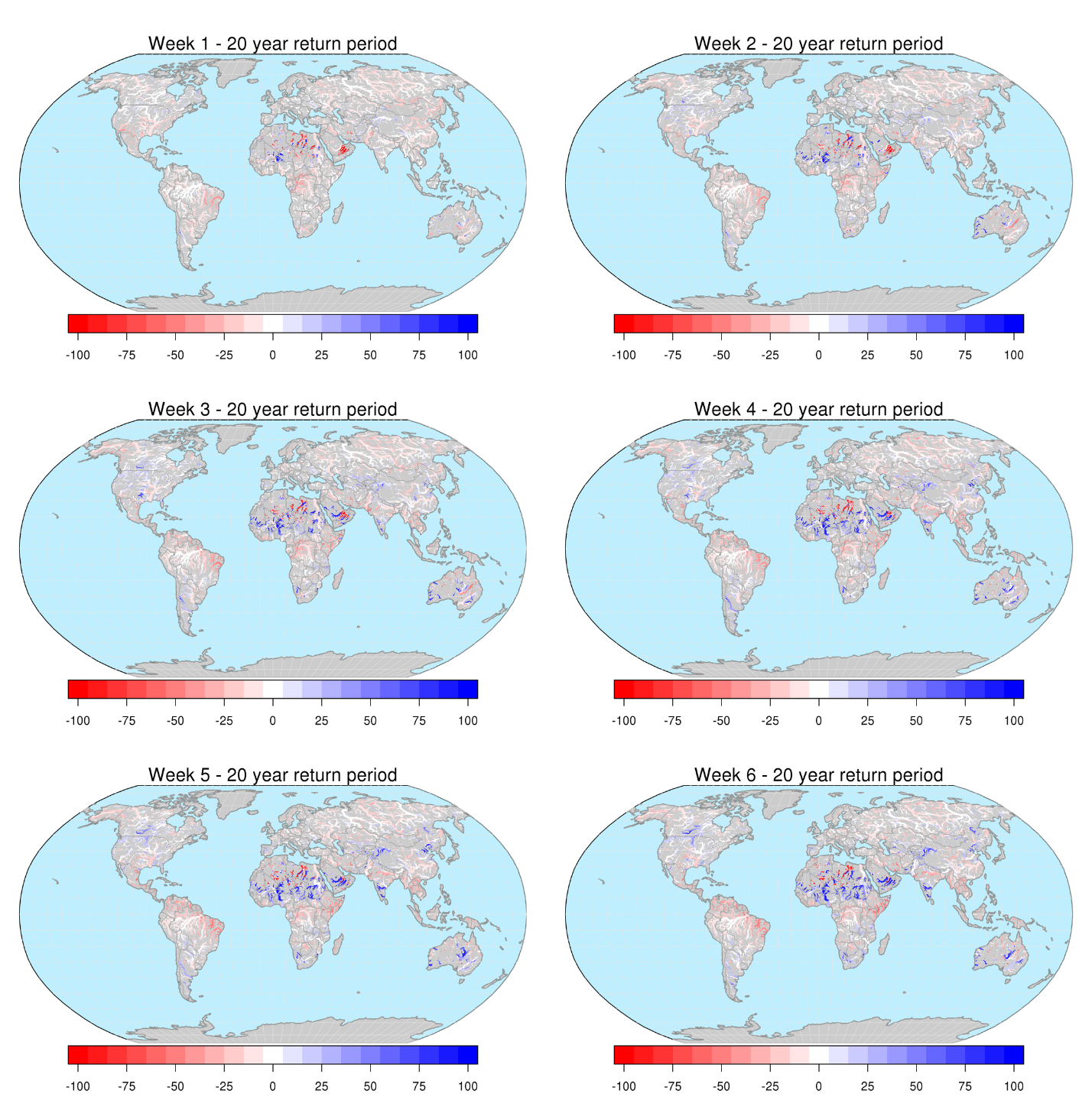
 Figure S7: Relative difference (in percent) in the 20-year threshold maps between reforecasts and ERA5, for forecast range between 1 and 6 weeks. Only river sections with upstream area larger than 10,000 km^2^ are shown, for easier interpretation of the plots.
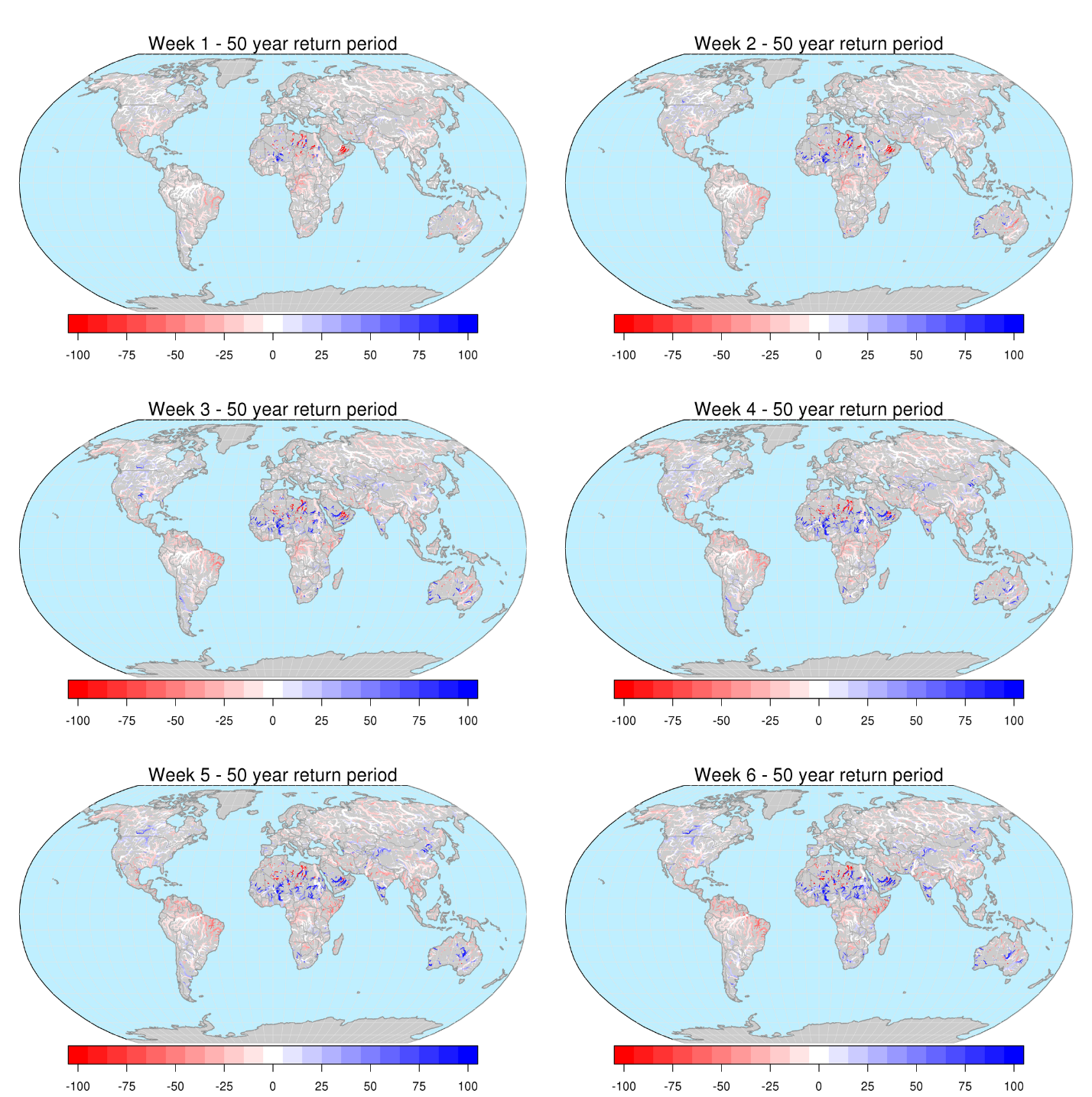
 Figure S8: Relative difference (in percent) in the 50-year threshold maps between reforecasts and ERA5, for forecast range between 1 and 6 weeks. Only river sections with upstream area larger than 10,000 km^2^ are shown, for easier interpretation of the plots.
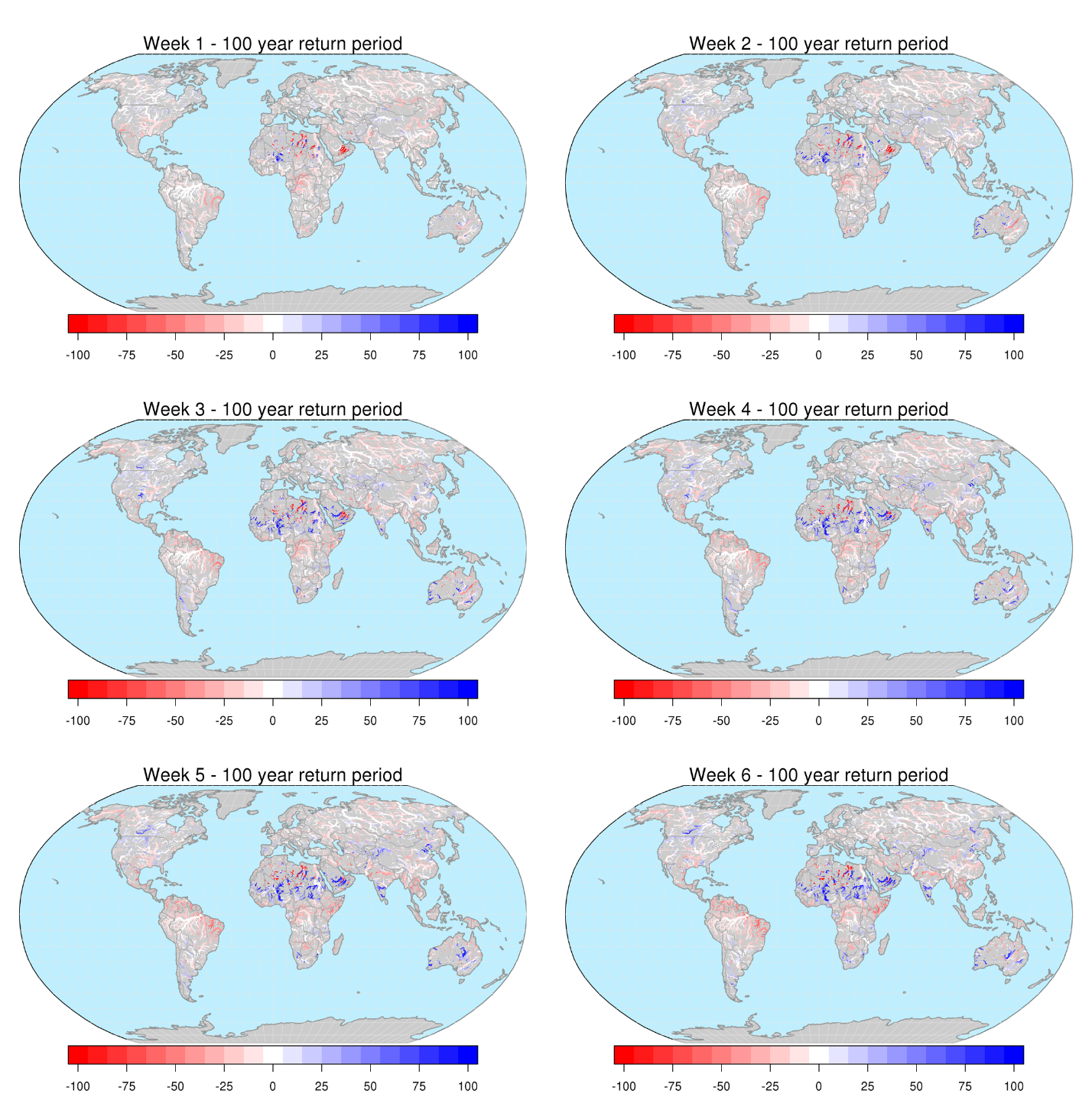
 Figure S9: Relative difference (in percent) in the 100-year threshold maps between reforecasts and ERA5, for forecast range between 1 and 6 weeks. Only river sections with upstream area larger than 10,000 km^2^ are shown, for easier interpretation of the plots.
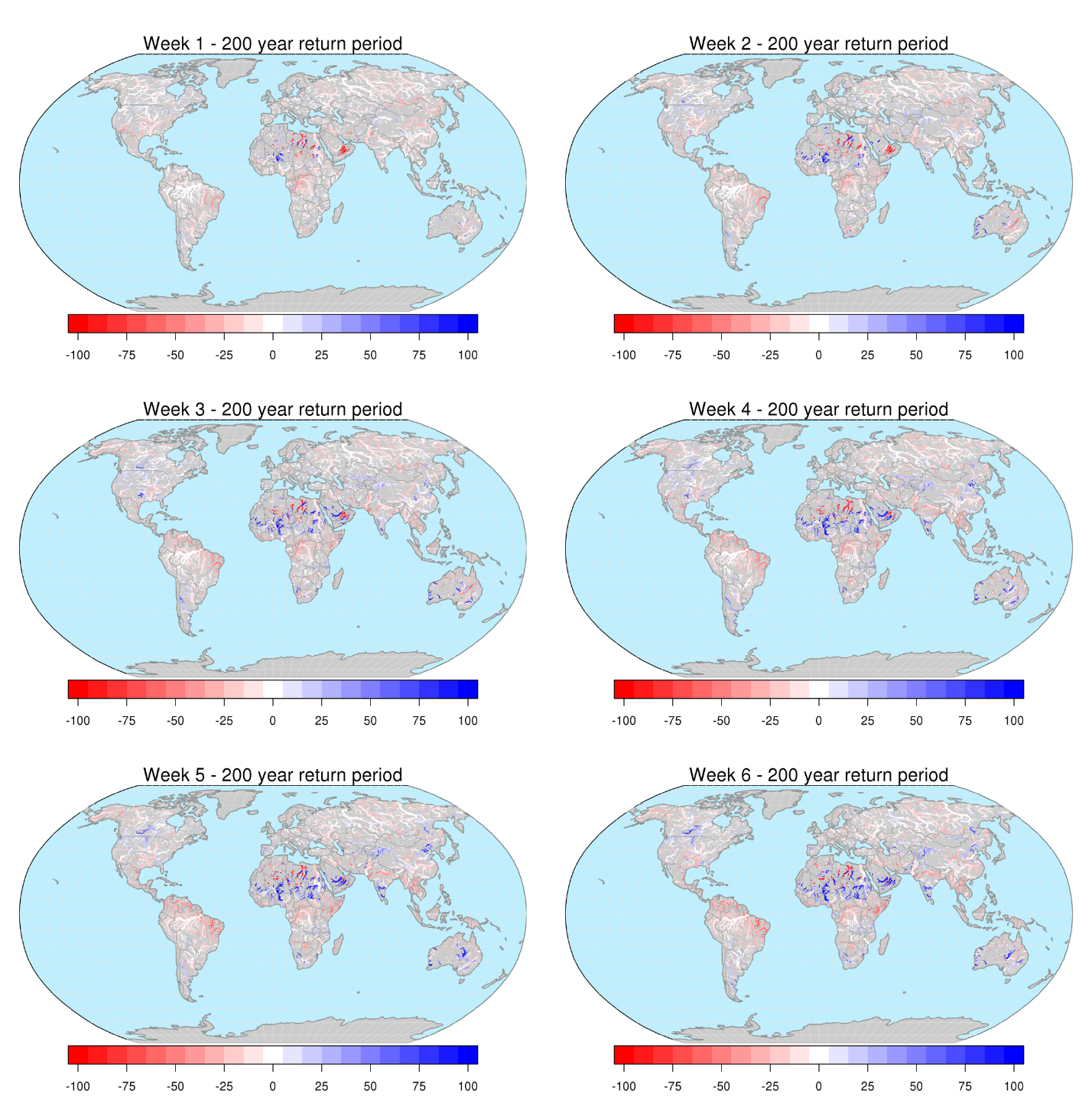
 Figure S10: Relative difference (in percent) in the 200-year threshold maps between reforecasts and ERA5, for forecast range between 1 and 6 weeks. Only river sections with upstream area larger than 10,000 km^2^ are shown, for easier interpretation of the plots.
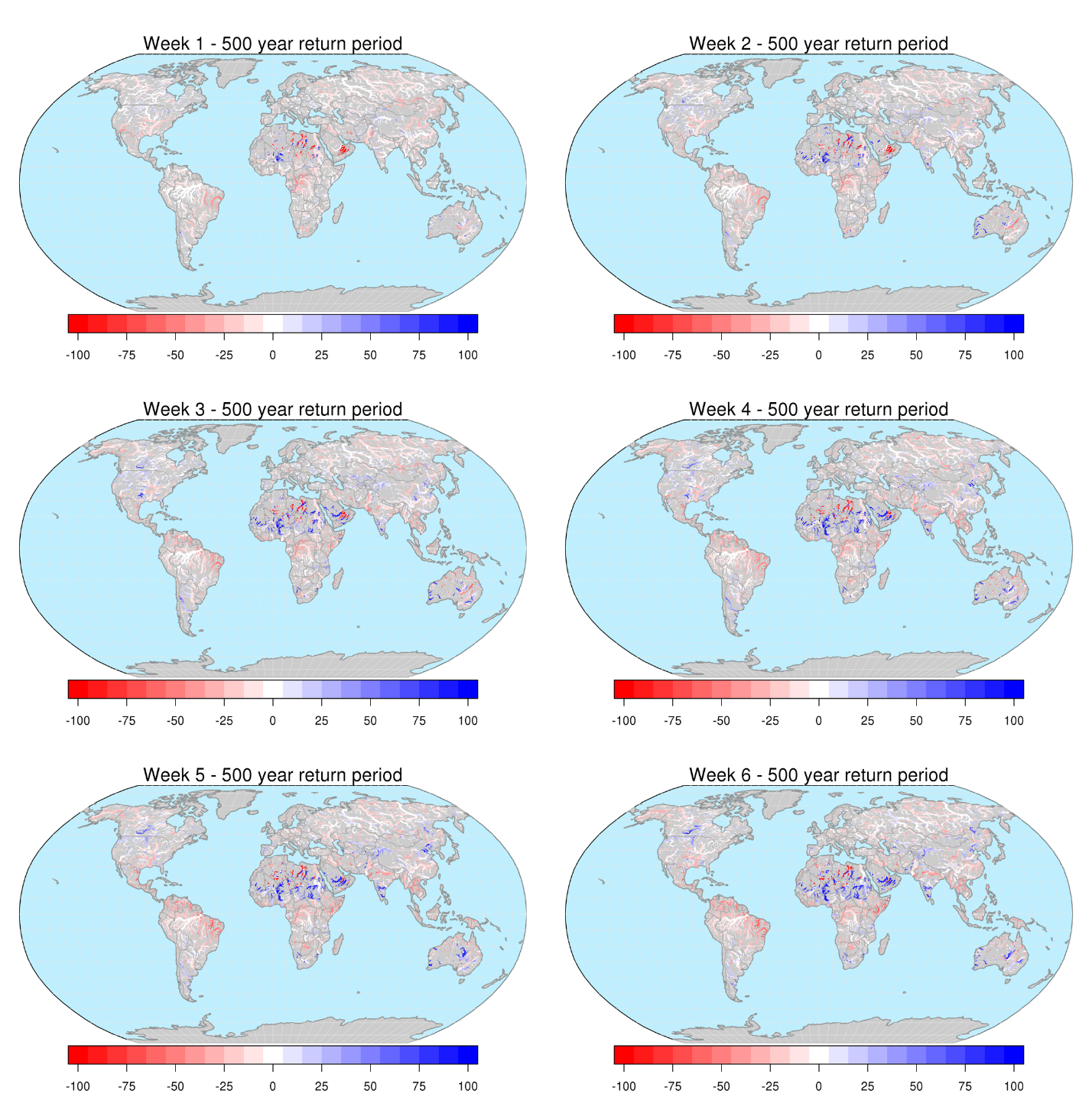
 Figure S11: Relative difference (in percent) in the 500-year threshold maps between reforecasts and ERA5, for forecast range between 1 and 6 weeks. Only river sections with upstream area larger than 10,000 km^2^ are shown, for easier interpretation of the plots.


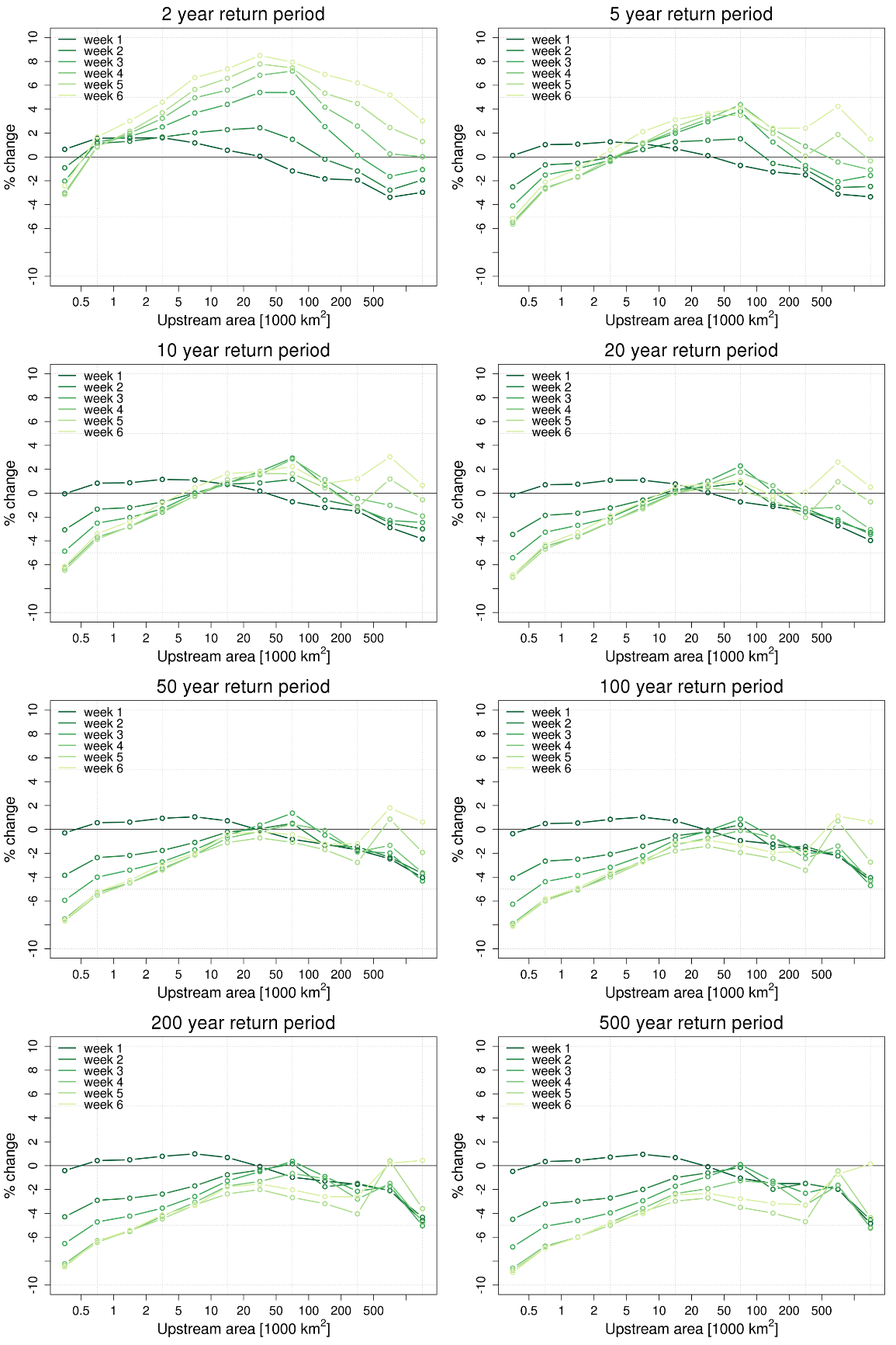


Figure S12: Relative difference (in percent) in threshold values between reforecasts and ERA5, for flood return periods between 2 and 500 years. Lines show median values for each class of upstream area for forecast range between 1 and 6 weeks.
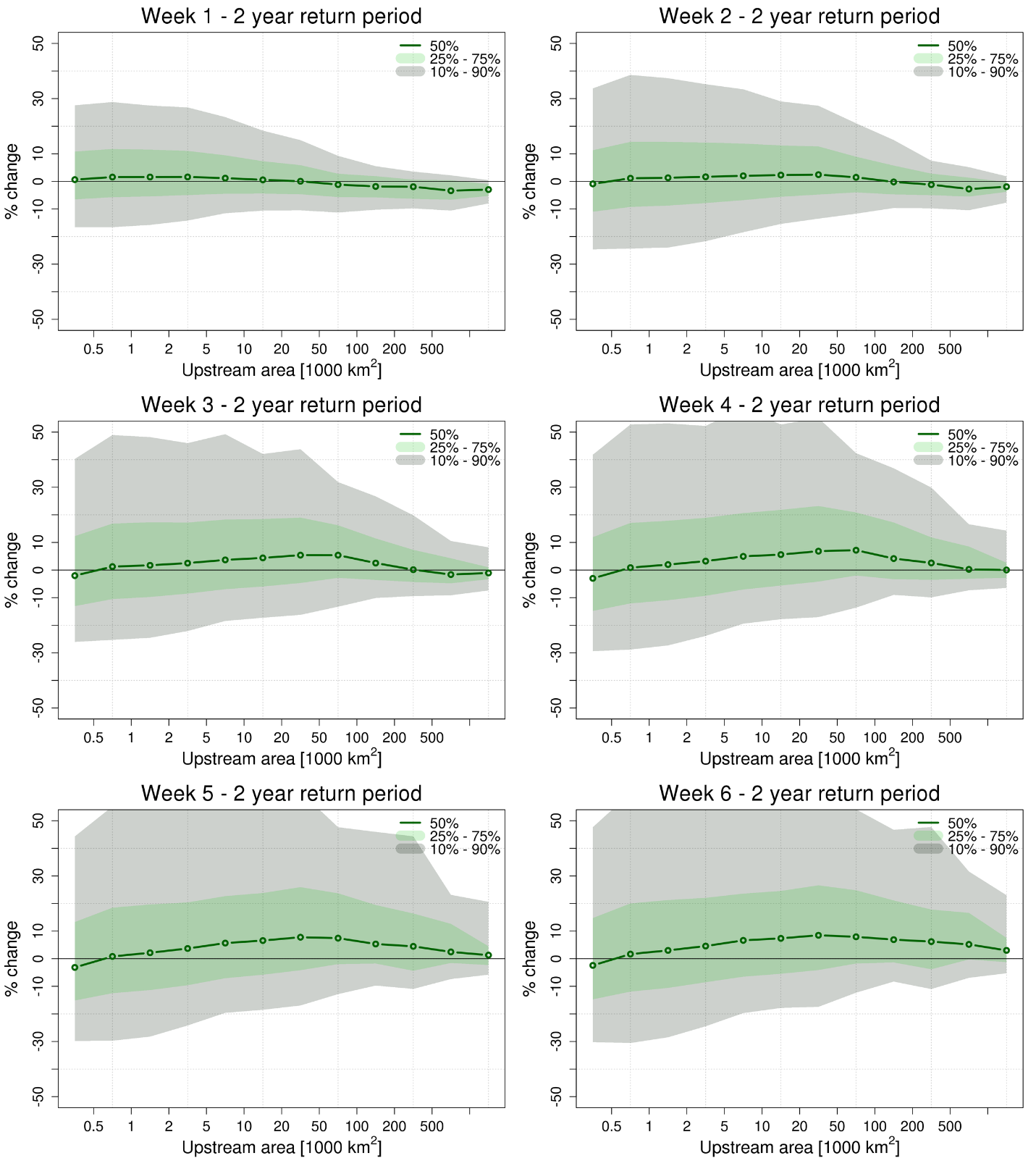
 Figure S13: Relative difference (in percent) in the 2-year threshold values between reforecasts and ERA5, for forecast range between 1 and 6 weeks. Median values for each class of upstream area are shown together with the 25%-75% range (in green) and the 10%-90% range (in grey).


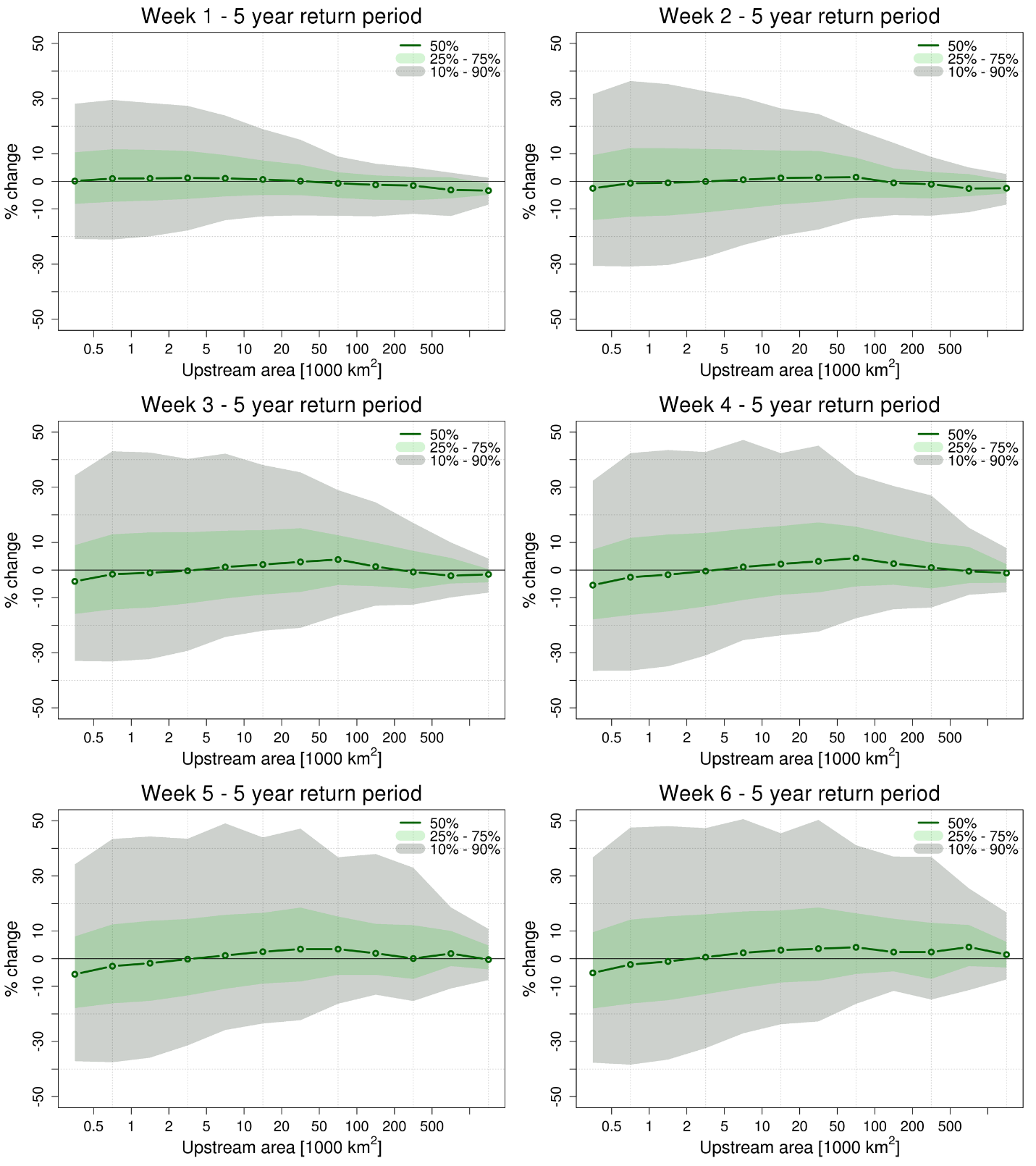
 Figure S14: Relative difference (in percent) in the 5-year threshold values between reforecasts and ERA5, for forecast range between 1 and 6 weeks. Median values for each class of upstream area are shown together with the 25%-75% range (in green) and the 10%-90% range (in grey).
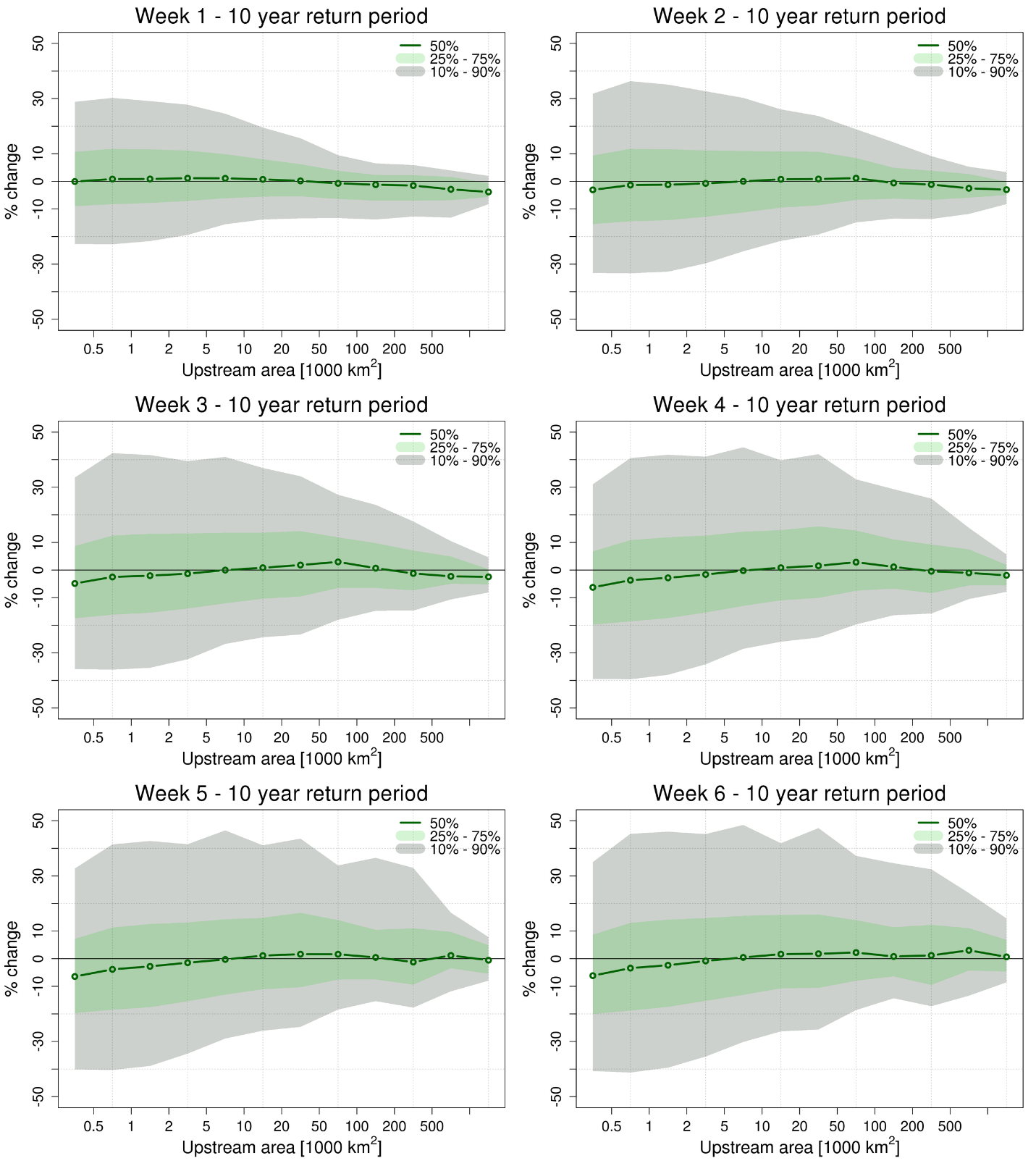
 Figure S15: Relative difference (in percent) in the 10-year threshold values between reforecasts and ERA5, for forecast range between 1 and 6 weeks. Median values for each class of upstream area are shown together with the 25%-75% range (in green) and the 10%-90% range (in grey).
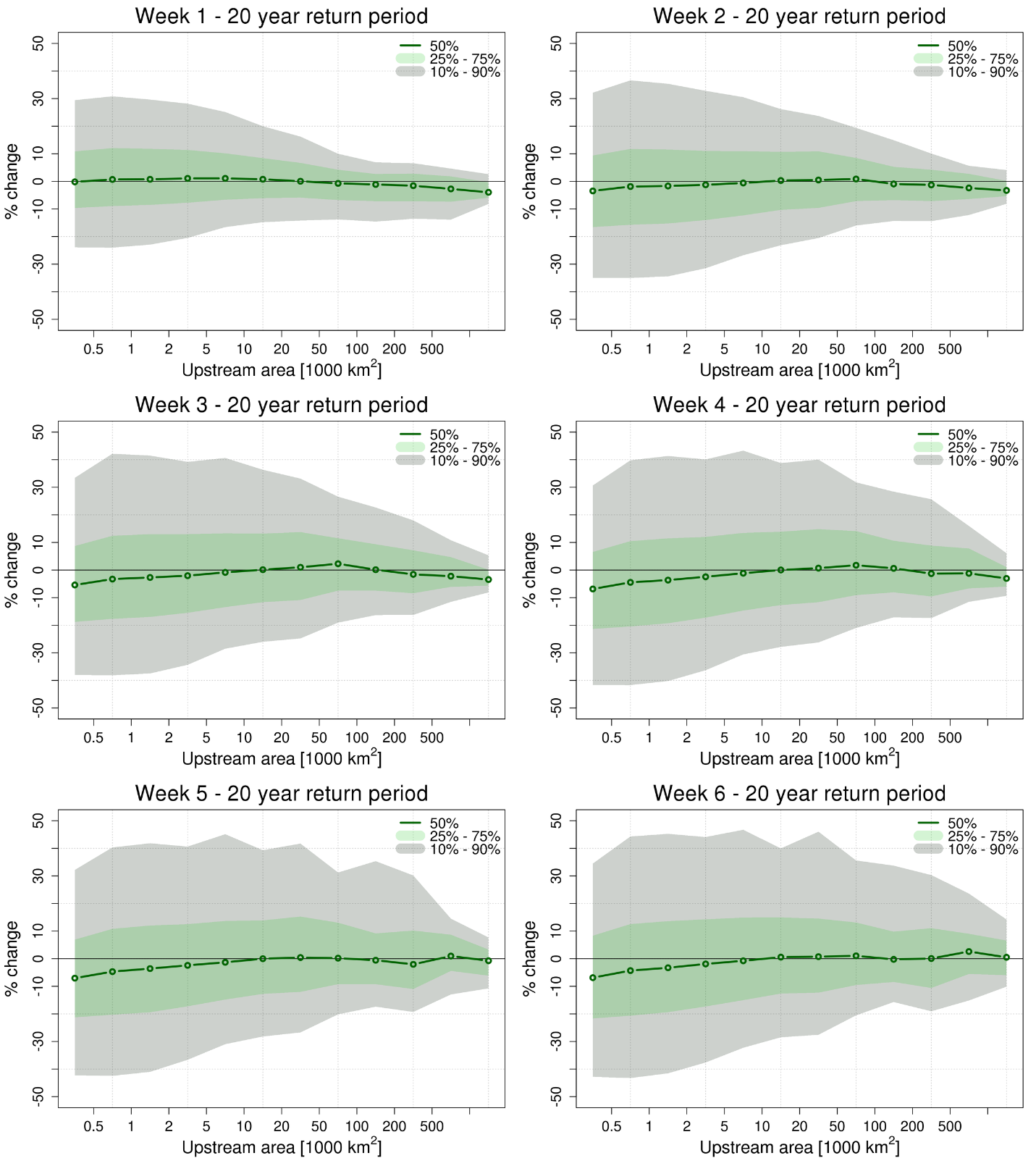
 Figure S16: Relative difference (in percent) in the 20-year threshold values between reforecasts and ERA5, for forecast range between 1 and 6 weeks. Median values for each class of upstream area are shown together with the 25%-75% range (in green) and the 10%-90% range (in grey).


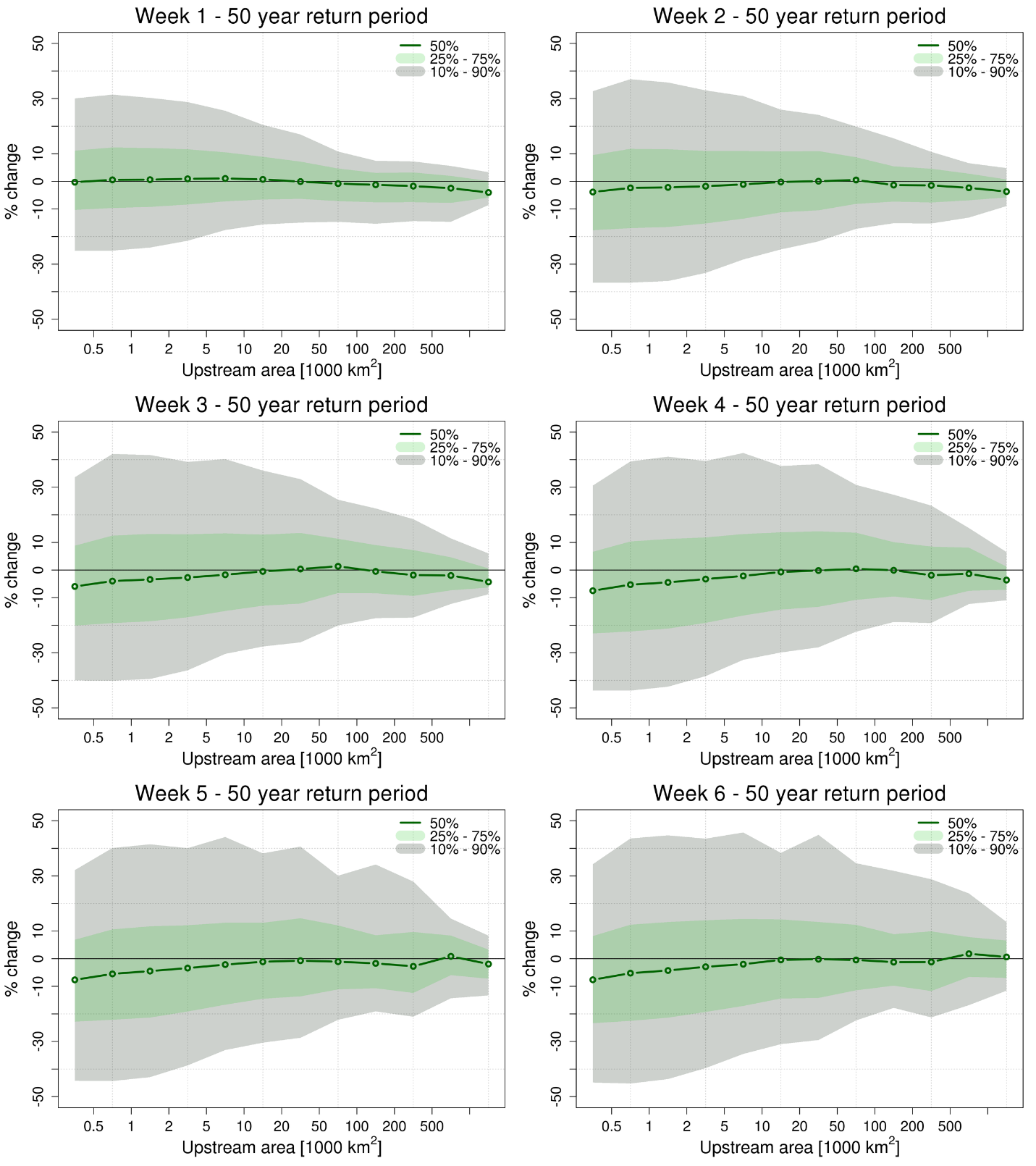


Figure S17: Relative difference (in percent) in the 50-year threshold values between reforecasts and ERA5, for forecast range between 1 and 6 weeks. Median values for each class of upstream area are shown together with the 25%-75% range (in green) and the 10%-90% range (in grey).
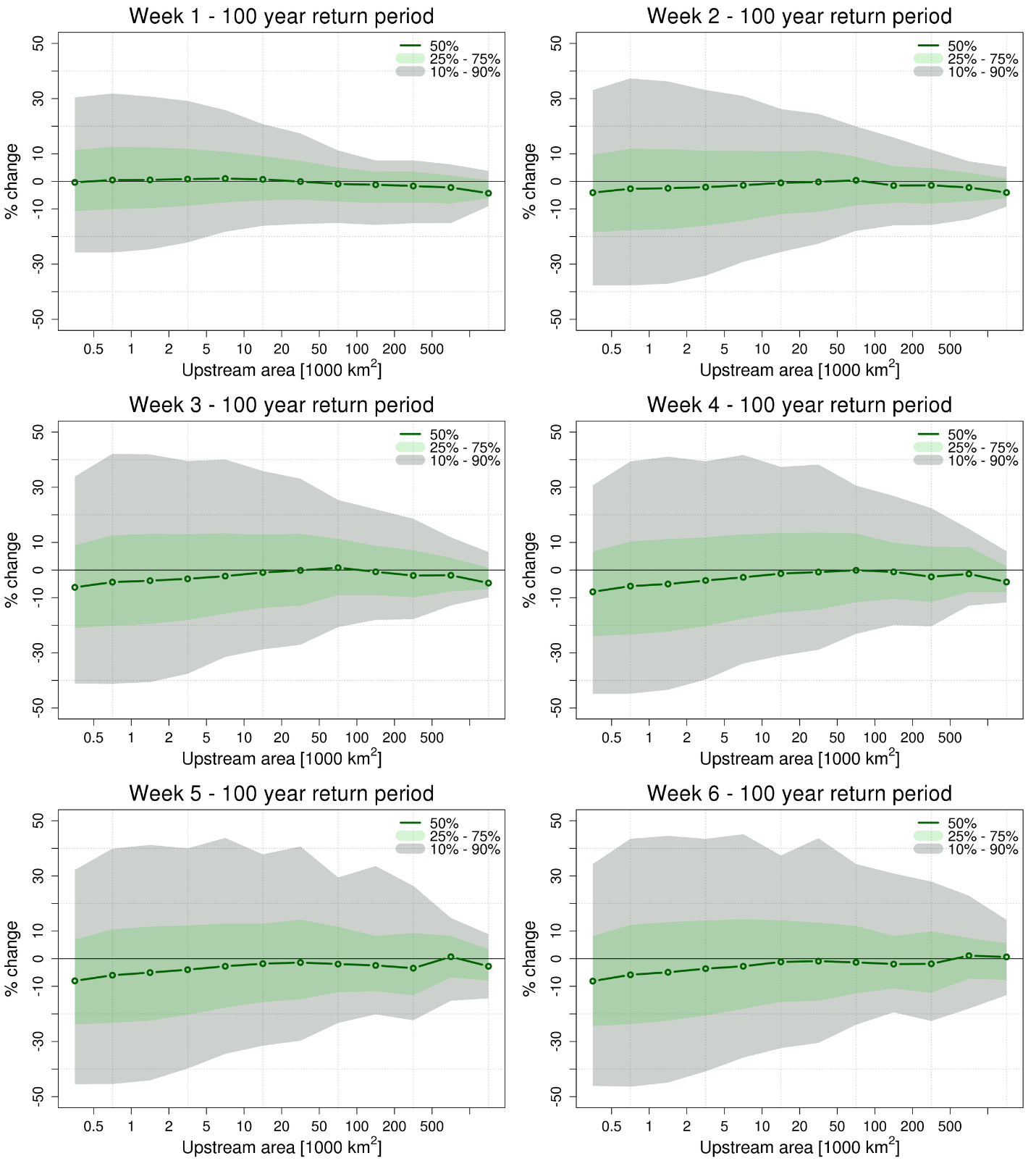
 Figure S18: Relative difference (in percent) in the 100-year threshold values between reforecasts and ERA5, for forecast range between 1 and 6 weeks. Median values for each class of upstream area are shown together with the 25%-75% range (in green) and the 10%-90% range (in grey).
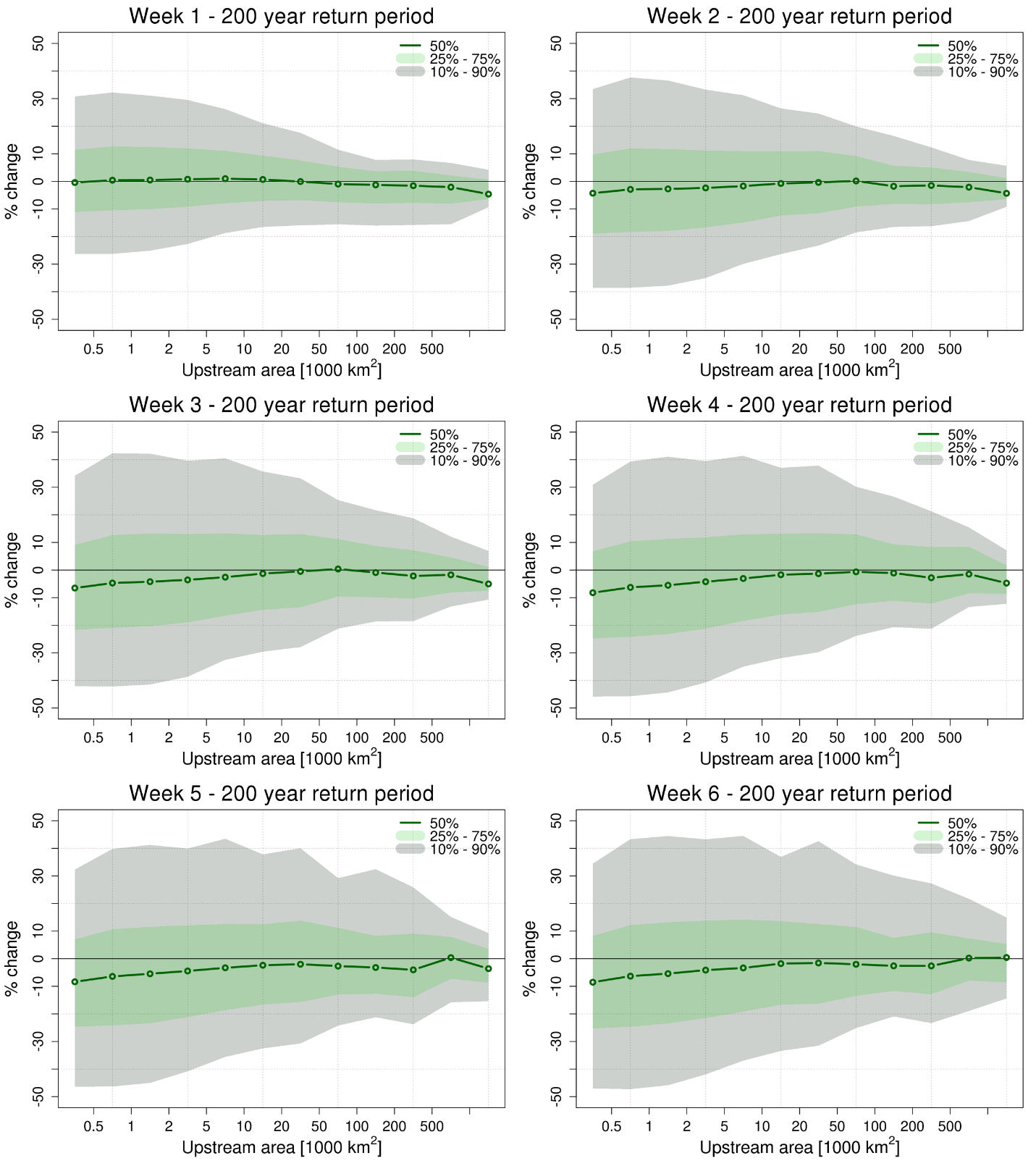
 Figure S19: Relative difference (in percent) in the 200-year threshold values between reforecasts and ERA5, for forecast range between 1 and 6 weeks. Median values for each class of upstream area are shown together with the 25%-75% range (in green) and the 10%-90% range (in grey).
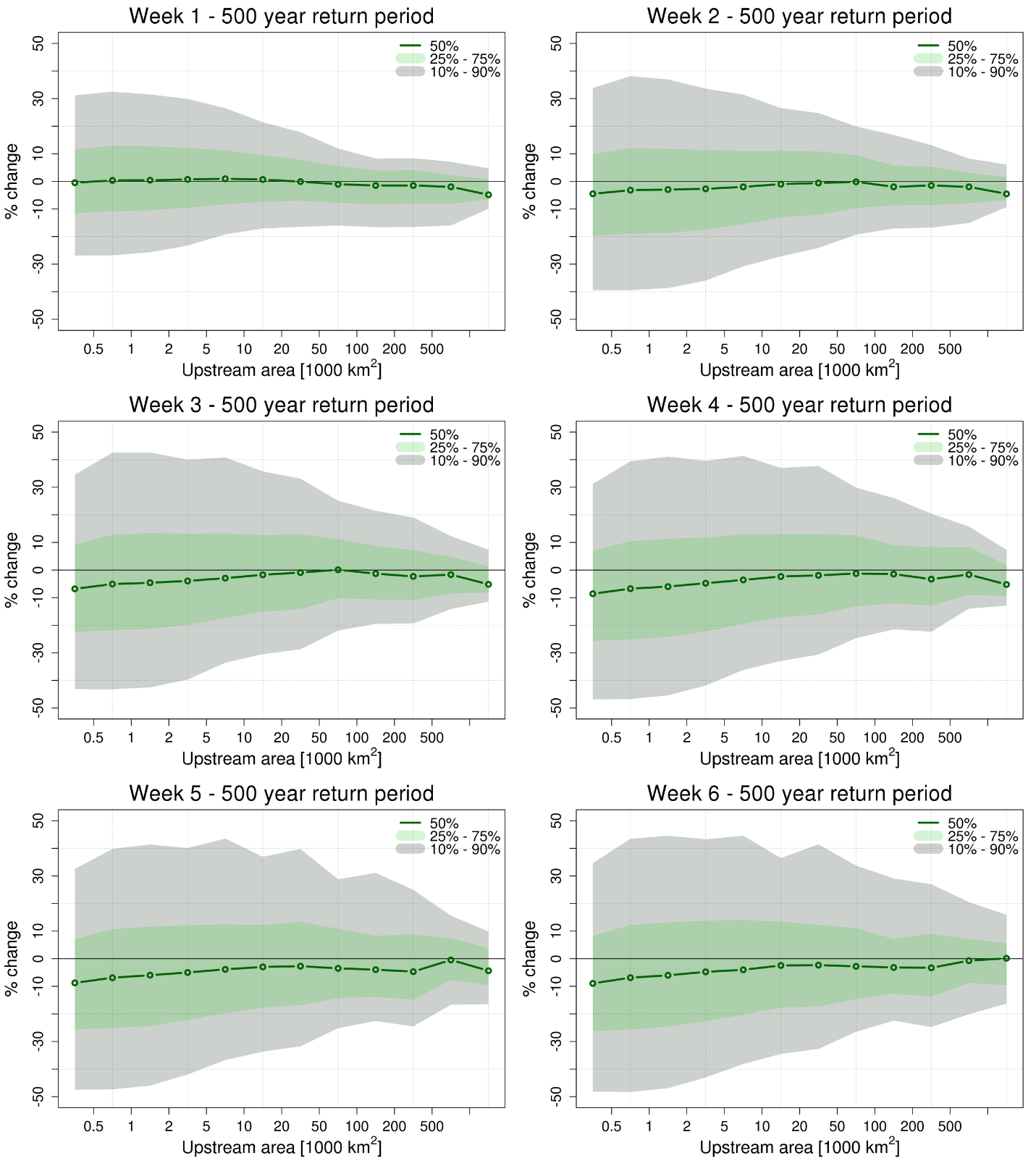
 Figure S20: Relative difference (in percent) in the 500-year threshold values between reforecasts and ERA5, for forecast range between 1 and 6 weeks. Median values for each class of upstream area are shown together with the 25%-75% range (in green) and the 10%-90% range (in grey).


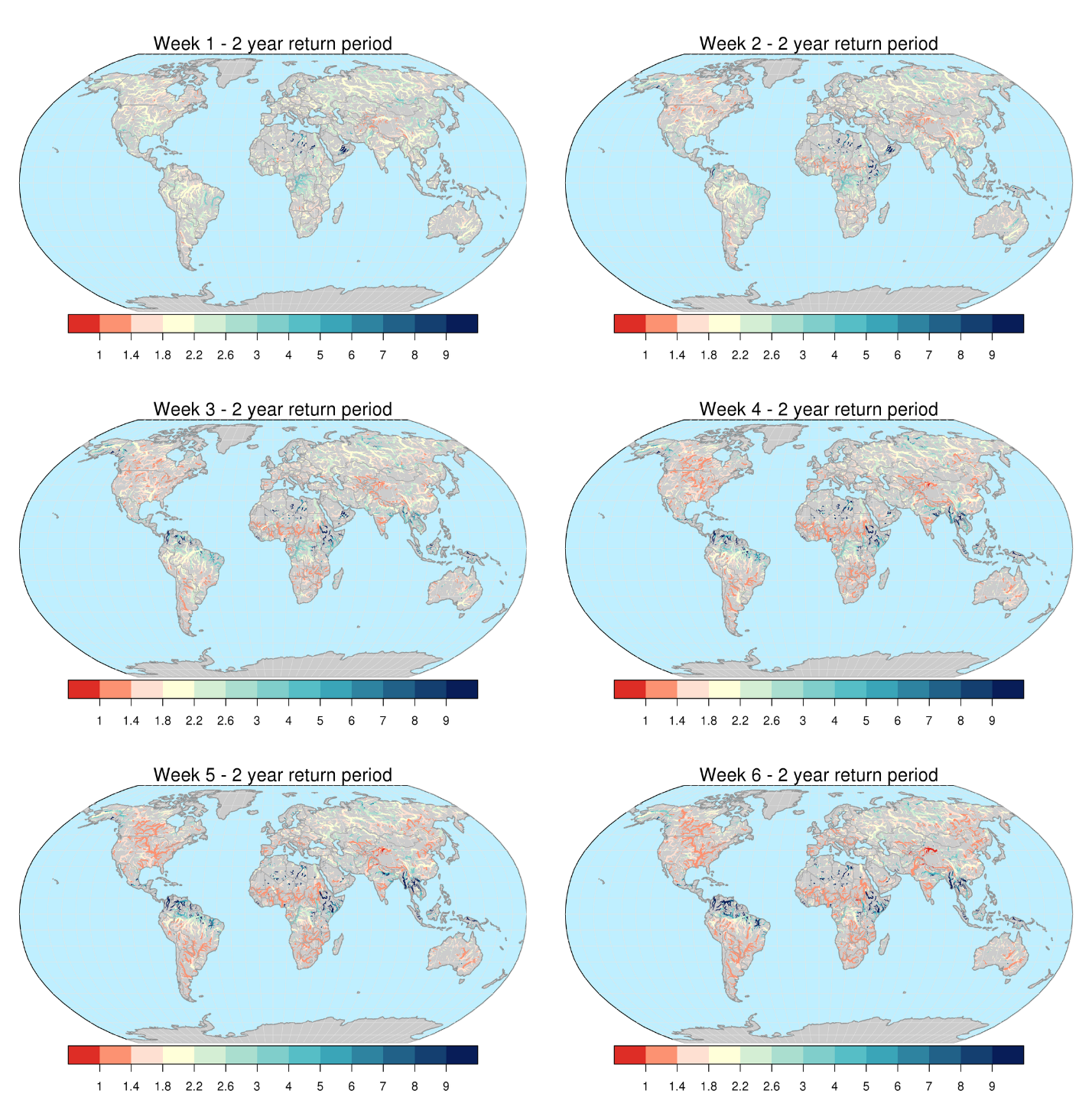
 Figure S21: Return period of the ERA5 2-year discharge threshold map, obtained using the extreme value distributions of reforecasts-driven simulations, for forecast range between 1 and 6 weeks. Only river sections with upstream area larger than 10,000 km^2^ are shown, for easier interpretation of the plots.
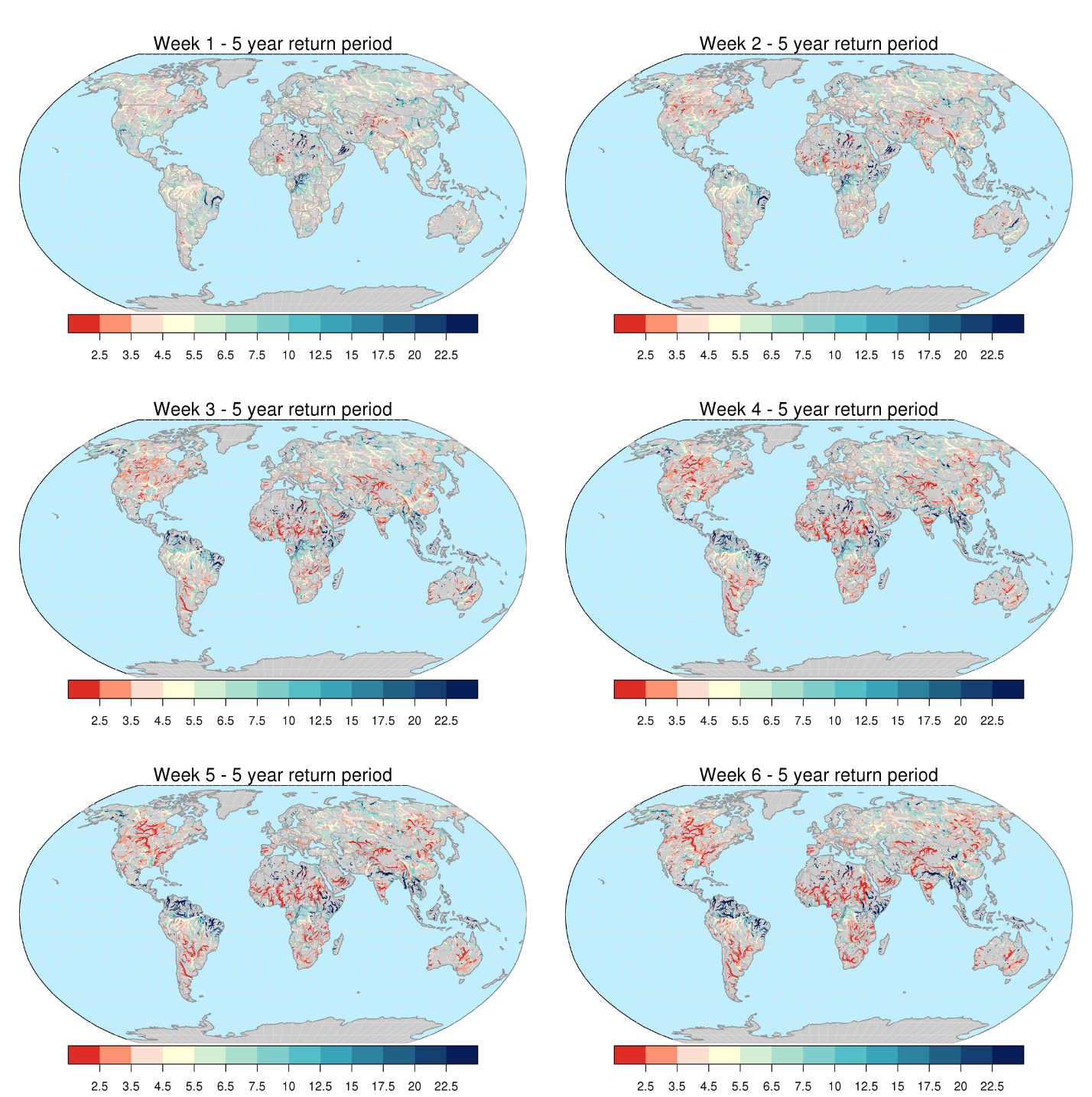
 Figure S22: Return period of the ERA5 5-year discharge threshold map, obtained using the extreme value distributions of reforecasts-driven simulations, for forecast range between 1 and 6 weeks. Only river sections with upstream area larger than 10,000 km^2^ are shown, for easier interpretation of the plots.
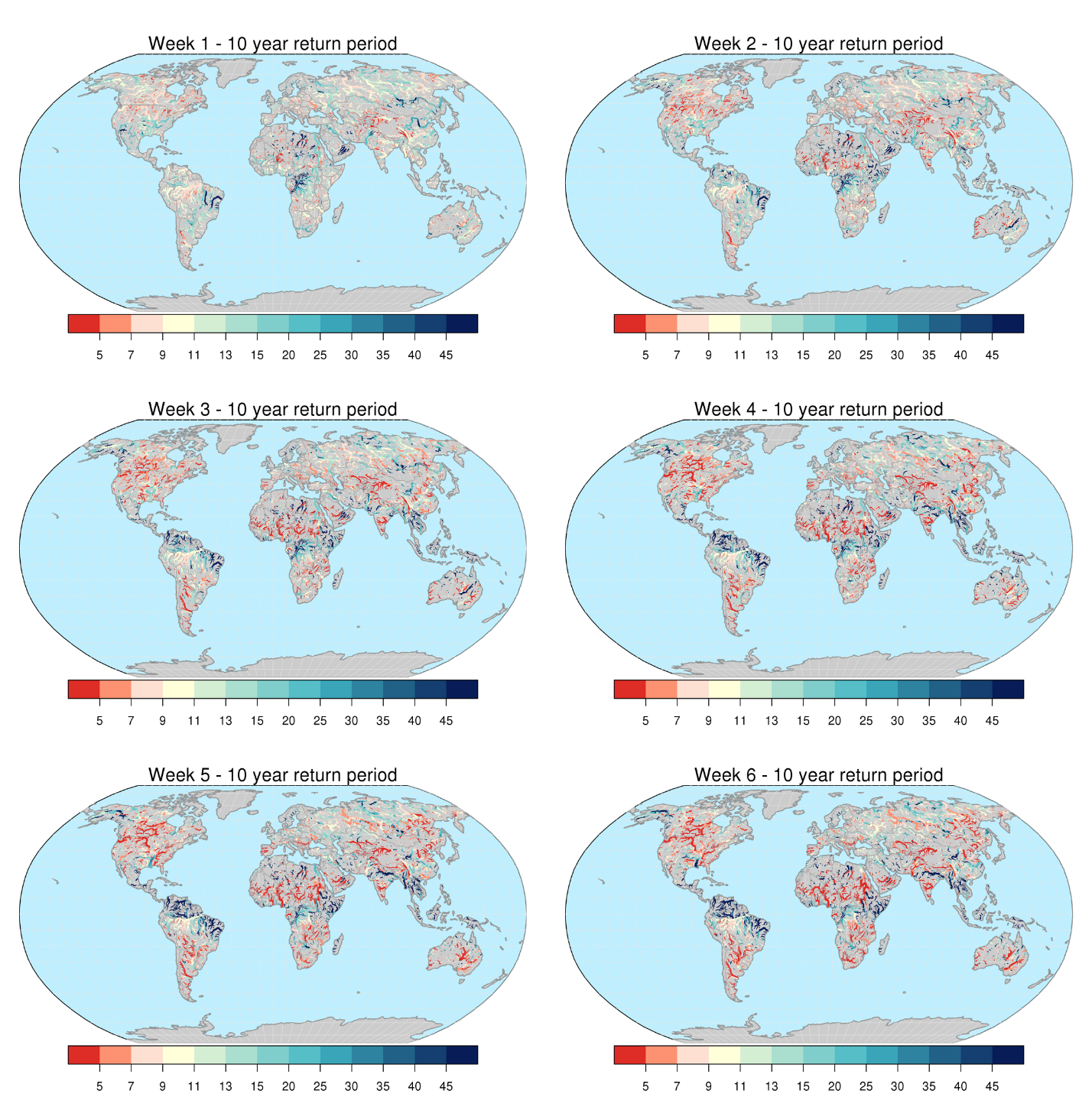
 Figure S23: Return period of the ERA5 10-year discharge threshold map, obtained using the extreme value distributions of reforecasts-driven simulations, for forecast range between 1 and 6 weeks. Only river sections with upstream area larger than 10,000 km^2^ are shown, for easier interpretation of the plots.
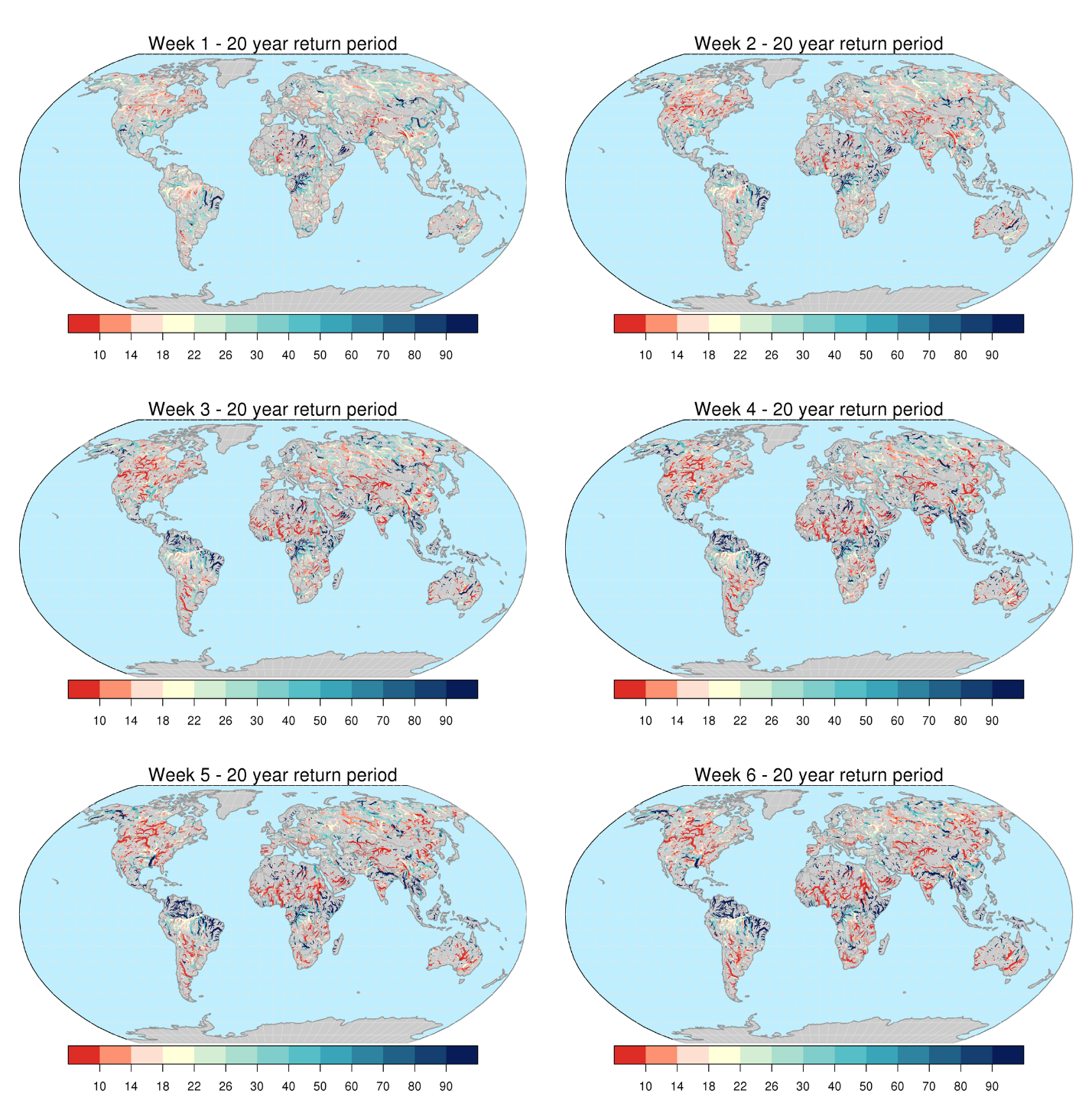
 Figure S24: Return period of the ERA5 20-year discharge threshold map, obtained using the extreme value distributions of reforecasts-driven simulations, for forecast range between 1 and 6 weeks. Only river sections with upstream area larger than 10,000 km^2^ are shown, for easier interpretation of the plots.
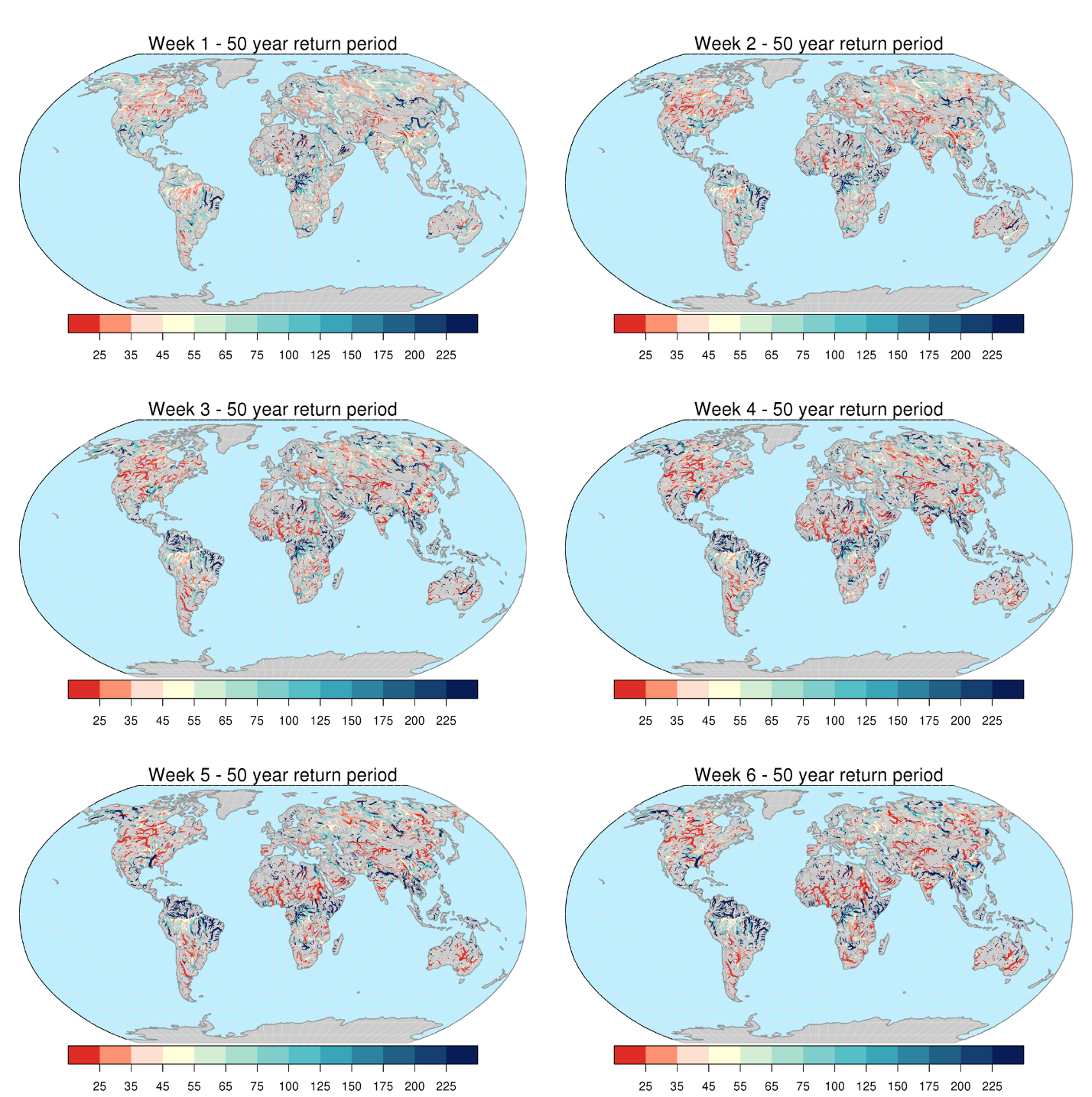
 Figure S25: Return period of the ERA5 50-year discharge threshold map, obtained using the extreme value distributions of reforecasts-driven simulations, for forecast range between 1 and 6 weeks. Only river sections with upstream area larger than 10,000 km^2^ are shown, for easier interpretation of the plots.
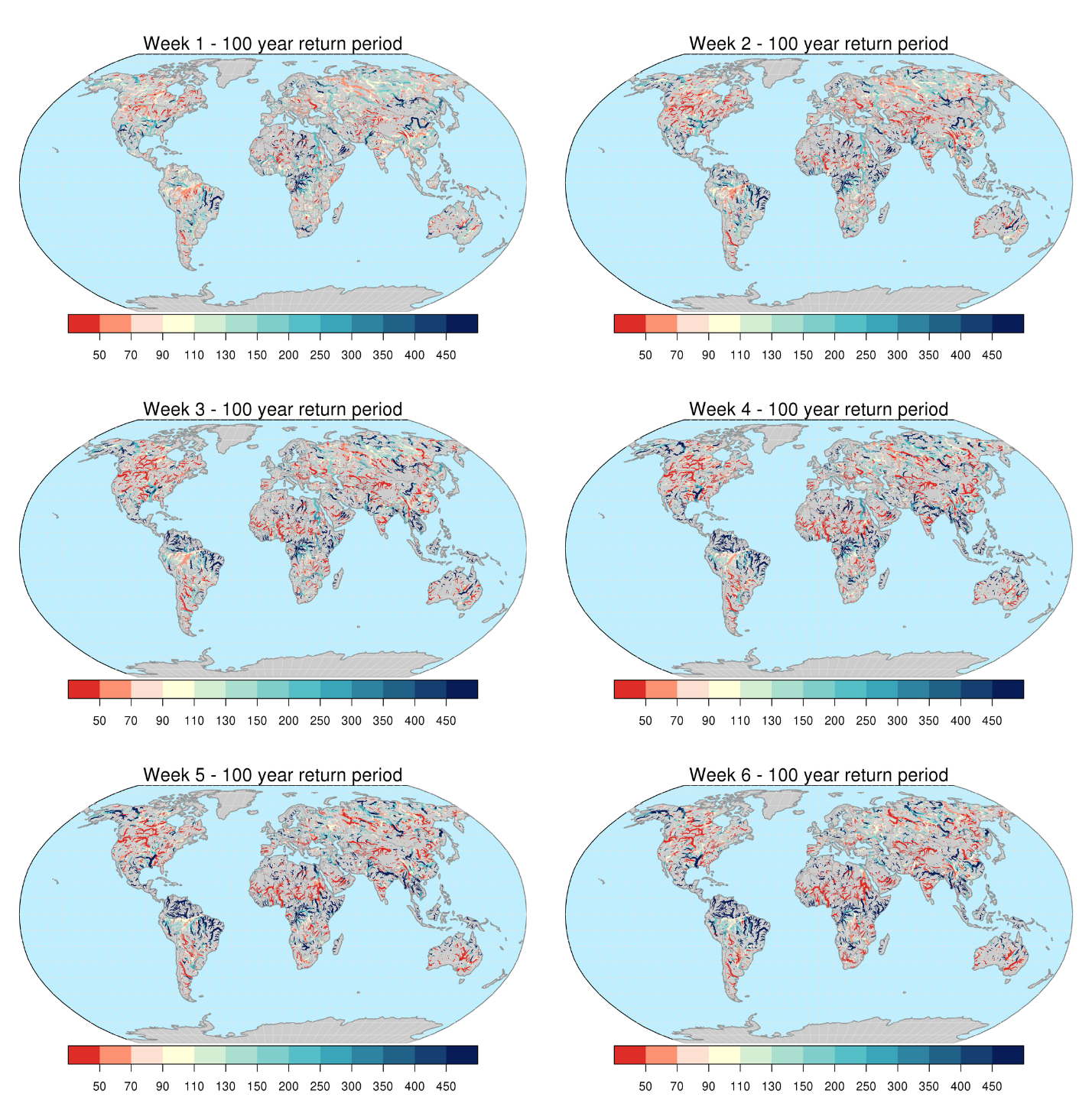
 Figure S26: Return period of the ERA5 100-year discharge threshold map, obtained using the extreme value distributions of reforecasts-driven simulations, for forecast range between 1 and 6 weeks. Only river sections with upstream area larger than 10,000 km^2^ are shown, for easier interpretation of the plots.
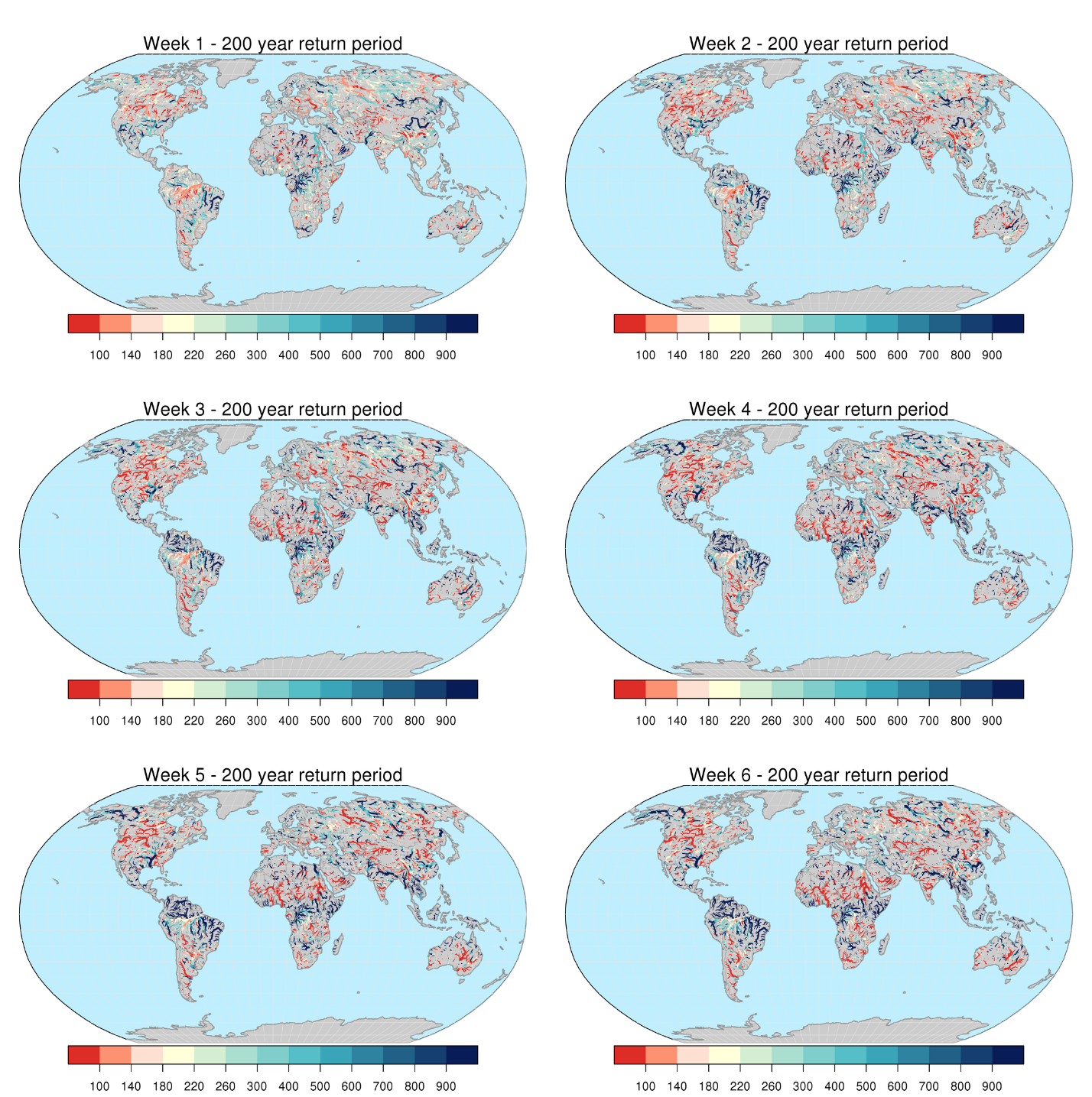
 Figure S27: Return period of the ERA5 200-year discharge threshold map, obtained using the extreme value distributions of reforecasts-driven simulations, for forecast range between 1 and 6 weeks. Only river sections with upstream area larger than 10,000 km^2^ are shown, for easier interpretation of the plots.
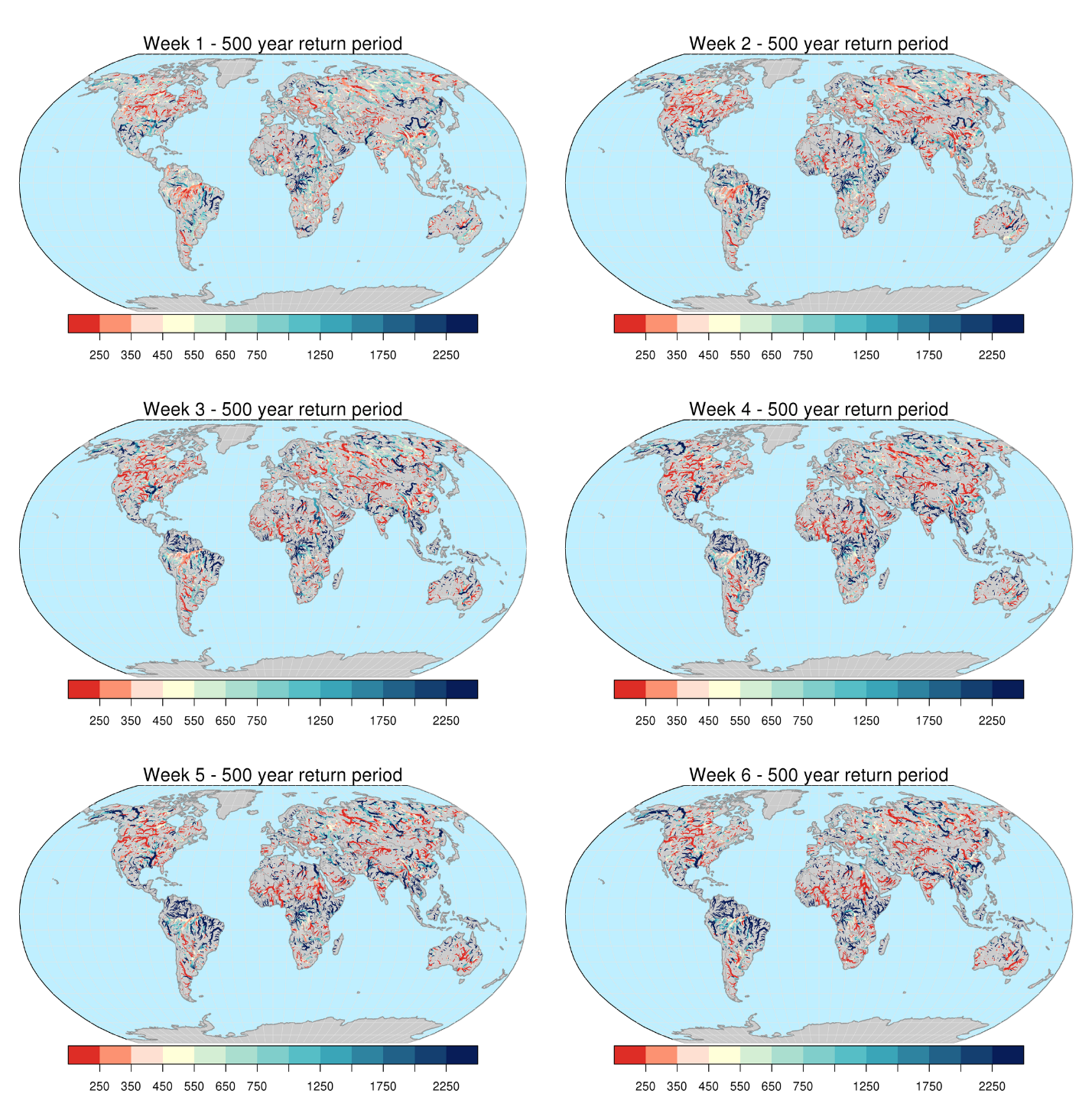
 Figure S28: Return period of the ERA5 500-year discharge threshold map, obtained using the extreme value distributions of reforecasts-driven simulations, for forecast range between 1 and 6 weeks. Only river sections with upstream area larger than 10,000 km^2^ are shown, for easier interpretation of the plots.


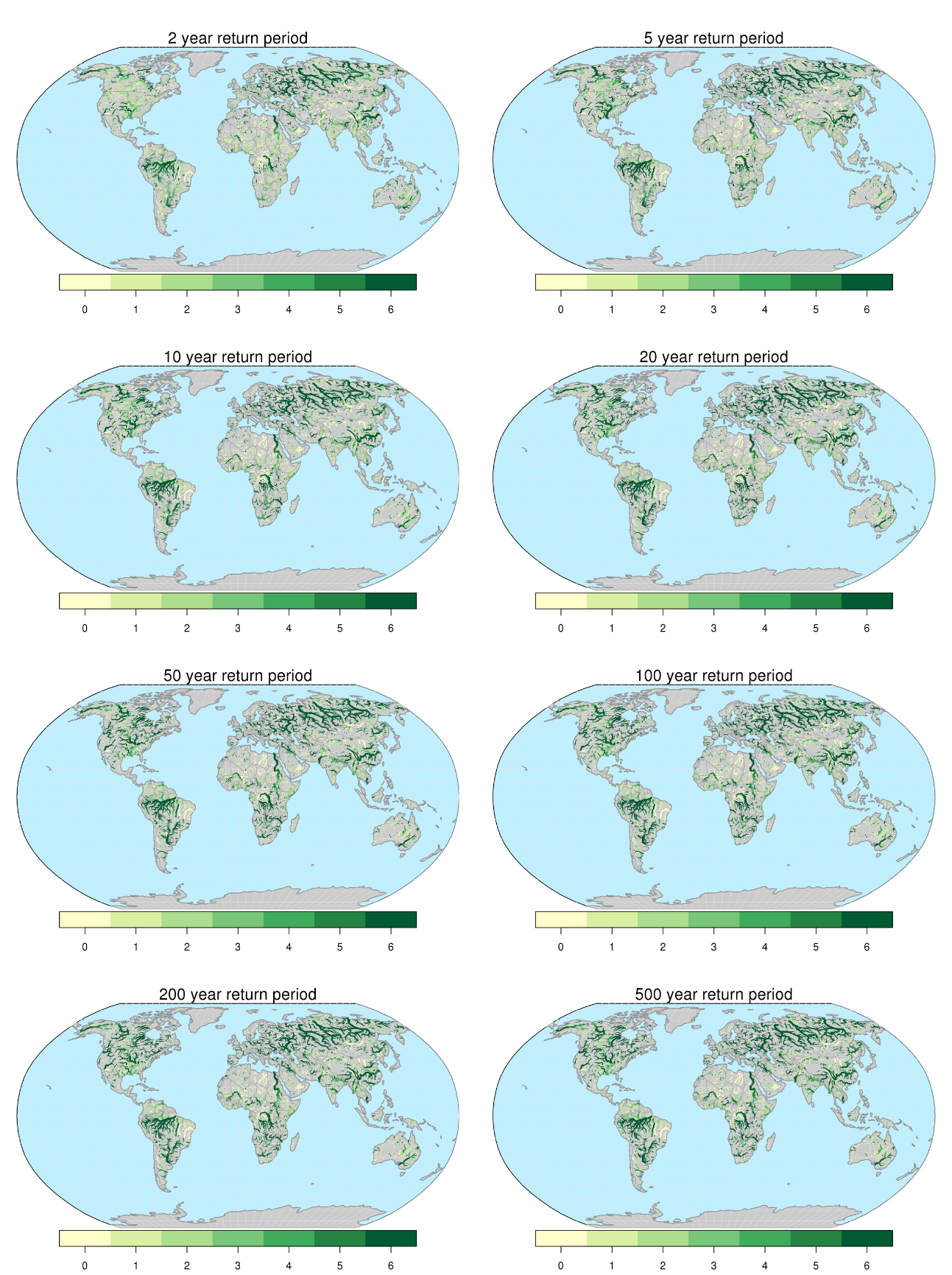


Figure S29: Number of weeks during which ERA5-based thresholds with 2- to 500-year return period are consistent with reforecast-based thresholds (i.e. within the 90% confidence bands).
